# Mechanisms of how sarcopenia affects functional outcomes in acute ischaemic stroke

**DOI:** 10.1093/braincomms/fcaf386

**Published:** 2025-11-11

**Authors:** Dong-Seok Gwak, Jinyong Chung, Dawid Schellingerhout, Hyerin Oh, Sang-Wuk Jeong, Ji Sung Lee, Dong-Eog Kim

**Affiliations:** Department of Neurology, Dongguk University Ilsan Hospital, Goyang 10326, Republic of Korea; National Priority Research Centre for Stroke, Goyang 10326, Republic of Korea; National Priority Research Centre for Stroke, Goyang 10326, Republic of Korea; Departments of Neuroradiology and Imaging Physics, University of Texas MD Anderson Cancer Centre, Houston, TX 77030, USA; National Priority Research Centre for Stroke, Goyang 10326, Republic of Korea; Department of Neurology, Dongguk University Ilsan Hospital, Goyang 10326, Republic of Korea; National Priority Research Centre for Stroke, Goyang 10326, Republic of Korea; Department of Biostatistics, Asan Medical Centre, Seoul 05505, Republic of Korea; Department of Neurology, Dongguk University Ilsan Hospital, Goyang 10326, Republic of Korea; National Priority Research Centre for Stroke, Goyang 10326, Republic of Korea

**Keywords:** ischaemic stroke, sarcopenia, muscle, prognosis, motor

## Abstract

Sarcopenia, a common geriatric condition characterized by the loss of skeletal muscle mass and function, can negatively affect functional outcomes in acute ischaemic stroke; however, the underlying mechanisms remain unclear. We hypothesized that sarcopenia affects post-stroke outcomes, mediated through its impact on dysphagia, early neurological deterioration, or post-stroke recovery, particularly in patients with motor deficits. To explore this hypothesis, we included 600 consecutive elderly (≥65 years) patients with acute (<1 week) ischaemic stroke and assessed: (i) the relationships sarcopenia has with dysphagia, early neurological deterioration and various phases of recovery after stroke (in-hospital, post-discharge [up to 3 months], and chronic [3 months to 1 year]) and (ii) whether either presenting symptoms or lesion locations modify the impact of sarcopenia on stroke outcomes. Temporal muscle thickness, a marker of sarcopenia, was measured by brain magnetic resonance imaging and dichotomized into low versus high temporal muscle thickness at the 25th percentile cut-off point. Logistic regression analysis, mediation analysis and statistical brain mapping were conducted. Mean age was 75.3 ± 6.1 years and 303 (50.5%) were male. Low temporal muscle thickness (<5.1 mm) was independently associated with dysphagia (adjusted odds ratio 1.89 [95% confidence interval 1.06–3.37], *P* = 0.03), early neurological deterioration (adjusted odds ratio 2.75 [95% confidence interval 1.61–4.71], *P* < 0.001) and post-discharge recovery (adjusted odds ratio 0.56 [95% confidence interval 0.34–0.94], *P* = 0.03) but not with in-hospital or chronic recovery. In addition, dysphagia, early neurological deterioration and post-discharge recovery were shown to mediate the association between low temporal muscle thickness and poor functional outcome (modified Rankin scale score ≥ 3) at 3 months, accounting for ∼45% of the total effect. Low temporal muscle thickness had a greater impact on stroke outcomes in patients with motor deficit, facial palsy, dysarthria, or pontine lesions. Furthermore, brain mapping revealed that low temporal muscle thickness had a stronger impact on functional outcomes in infarctions involving brain regions responsible for motor strength, planning, execution, and control: i.e. pallidum, fronto-pontine tract, parieto-occipito-temporo-pontine tract, middle cerebellar peduncle, and corticospinal tract. Sarcopenia leads to poor functional outcomes, probably due to its association with dysphagia, early neurological deterioration, and limited post-discharge recovery in elderly acute ischaemic stroke patients, particularly those with motor deficit, bulbar symptoms, or lesions involving pons and motor pathways. Understanding and identifying the mechanisms underlying sarcopenia-related effects on post-stroke outcomes may inform comprehensive, time-specific approaches to personalised management for this patient population.

## Teaser

Gwak *et al*. find that sarcopenia was associated with dysphagia, early neurological deterioration, and decreased post-discharge recovery (up to 3 months post-stroke) in acute ischaemic stroke, contributing to poor functional outcomes. Sarcopenia more strongly affects outcomes in patients with motor deficits, bulbar symptoms, or motor pathway lesions.

## Graphical Abstract

**Graphical Abstract:**
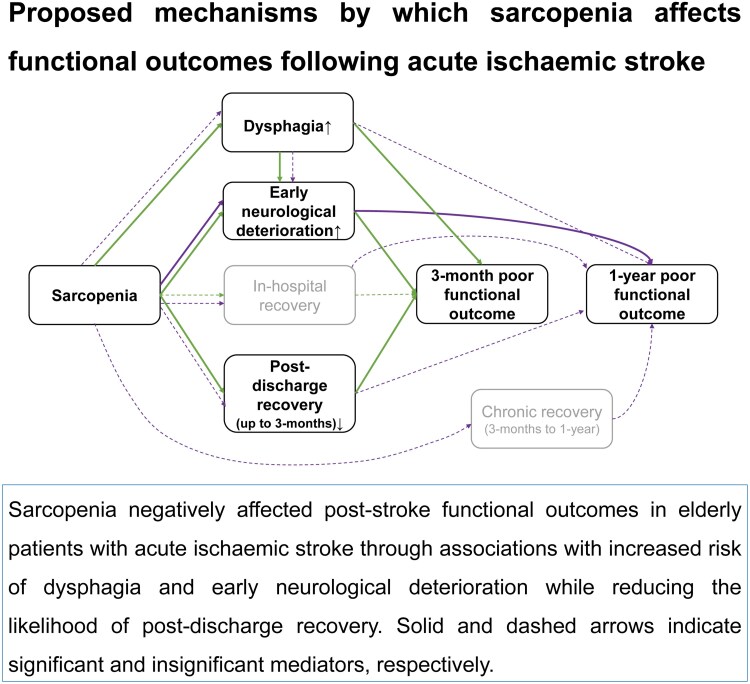

## Introduction

As the global population rapidly ages, it is vitally important to understand concurrent geriatric conditions in patients with cerebrovascular disease.^1^ Sarcopenia, the loss of skeletal muscle mass and function, is one of the geriatric conditions that leads to a decline in physical performance, increased risk of falls, and overall frailty.^2^ Approximately a quarter of patients with acute ischaemic stroke (AIS) have premorbid sarcopenia.^3^

Previous studies have shown that sarcopenia can negatively affect post-stroke functional outcomes (as reviewed in Supplementary Table 1).^4-14^ Several possible explanations have been proposed (Supplementary Table 1),^15-21^ yet the underlying mechanisms remain unclear. Prior research demonstrated an association between sarcopenia and dysphagia, which is linked to increased morbidity and mortality after stroke.^16,17,22^ However, the mediating role of dysphagia in sarcopenia’s effect on functional outcomes has not been explored. A recent study showed a relationship between sarcopenia and early neurological deterioration (END),^19^ but the detailed mechanism behind sarcopenia-related END has not been investigated. Moreover, most of these studies assessed sarcopenia by using either bioelectrical impedance analysis^4-8,16^ or the Strength, Assistance with walking, Rising from a chair, Climbing stairs and Falls questionnaire.^9,10,15,16,18,19^ These tests require cooperation from the subject, which can be difficult for stroke patients who are experiencing paralysis, cognitive impairments, or reduced consciousness. Thus, previous research using these tests excluded a high proportion (up to ∼70%) of eligible patients.

Temporal muscle thickness (TMT) has been shown to correlate with hand grip strength^23^ and skeletal muscle mass as measured with other methods,^20,24^ thus indicating TMT to be a reliable surrogate marker for sarcopenia. Moreover, TMT is an efficient imaging biomarker for sarcopenia in AIS,^25^ in which brain MRI or CT is routinely performed. There is great need to investigate TMT in terms of its impact on AIS care and related mechanisms, including: (i) initial dysphagia; (ii) END stratified by different causes; (iii) post-stroke recovery, and (iv) functional outcomes. Additionally, interactions with presenting symptoms and lesion locations should be studied, as the heterogeneity of stroke, driven by this variability, further complicates our understanding of the mechanisms linking sarcopenia to stroke outcomes.

In this study, considering that sarcopenia is characterized by a decline in the quality and quantity of skeletal muscle,^26^ we tested our hypothesis that sarcopenia could affect post-stroke outcomes, mediated through its impact on dysphagia, END, or post-stroke recovery, particularly in patients with motor deficits. We also conducted statistical and mapping analyses, with stratifications by presenting symptoms and lesion locations.

## Materials and methods

### Study population

We retrospectively analysed prospectively collected data from a single-centre stroke registry. From May 2011 to December 2015, a total of 1292 consecutive patients with ischaemic stroke who visited our centre within 7 days of stroke onset were screened. We included patients aged ≥65 years (*n* = 822). We excluded those with (i) pre-stroke modified Rankin Scale (mRS) score of two or more (*n* = 190); (ii) no or inadequate MRI data for measuring TMT (*n* = 8 and 1, respectively); (iii) follow-up loss at 3 months after the index stroke (*n* = 23) (Supplementary Fig. 1A). This study was performed in accordance with good clinical practices and the Declaration of Helsinki. The Institutional Review Board approved the study protocol (IRB protocol no. DUIH 2023-11-011). All participants gave written informed consent. This study was conducted in accordance with the Strengthening the Reporting of Observational Studies in Epidemiology statement.^27^

### Clinical data and outcome measurements

Our institutional stroke registry, a subset of the nationwide CRCS-K-NIH registry,^28^ prospectively collected National Institutes of Health Stroke Scale (NIHSS) scores at admission (including both total and itemised scores); pre-stroke mRS score and mRS score at discharge, 3 months, and 1 year after stroke, in accordance with a standardized protocol.^29^ Through a web-based education system (http://www.stroke-edu.or.kr/), we trained and certified physicians and nurses at our centre to assess NIHSS scores and mRS scores. We also collected demographic data; laboratory data; past medical history including risk factors for stroke such as hypertension, diabetes mellitus, hyperlipidaemia, coronary artery disease, atrial fibrillation and smoking history; and AIS lesion location. Stroke subtypes were determined using a validated MRI-based algorithm built on the Trial of Org 10172 in Acute Stroke Treatment (TOAST) criteria.^30^ Dysphagia was screened at admission and defined as either tube-dependent feeding or restricted total oral intake, corresponding to the Functional Oral Intake Scale of 1–6.^16,17^ END was deﬁned as any new neurological symptoms/signs or any neurological worsening within 3 weeks after stroke onset with (i) an increment in the total NIHSS score of ≥2 points; (ii) an increment in the consciousness score (1a–1c) of NIHSS score ≥1; (iii) an increment in the motor score (5a–6b) of NIHSS score ≥1; or (iv) any new neurological deﬁcit.^31^ END was categorized into stroke progression, stroke recurrence, transient ischaemic attack, symptomatic haemorrhagic transformation (with ≥4 points increase in NIHSS score), unknown and others. Post-stroke recovery periods were divided into three periods: (i) in-hospital recovery defined as an improvement of ≥4 points or ≥40% in a NIHSS score from admission to discharge^32^; (ii) post-discharge recovery defined as a reduction in the 3-month mRS score compared to the discharge mRS score (3-month mRS—discharge mRS < 0), excluding patients with a discharge mRS score of either 0 (asymptomatic) or 6 (dead)^31^ and (iii) chronic recovery defined as lower 1-year mRS score compared with 3-month mRS score after excluding patients whose 3-month mRS score was 0 or 6.^33,34^

### Measuring TMT and defining sarcopenia

**Figure 1:**
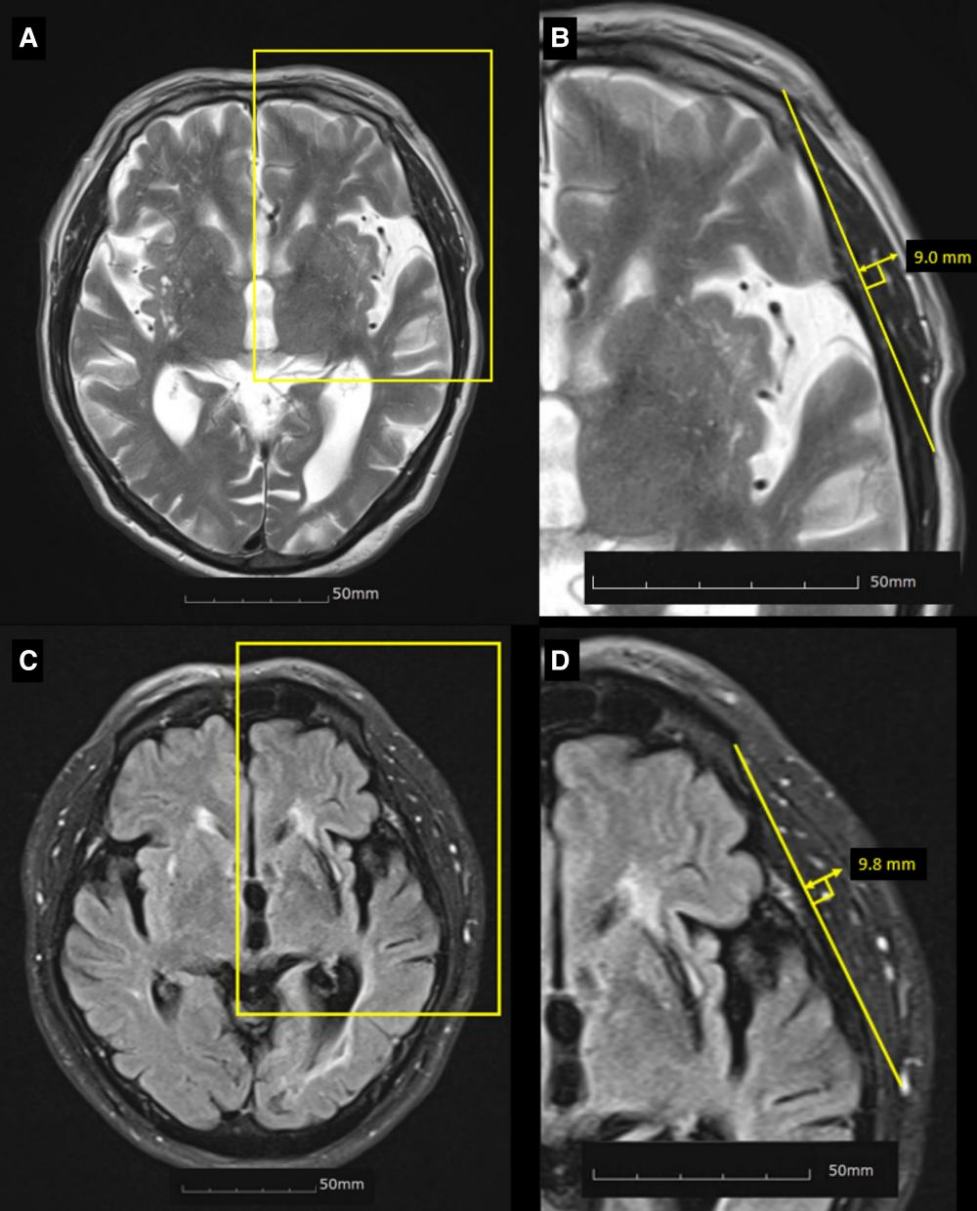
**Measuring TMT.** TMT was measured on the axial plane of either **(A and B)** T2-weighted or **(C and D)** FLAIR images, which were oriented parallel to the anterior commissure–posterior commissure line. **(A, C)** TMT was assessed at the orbital roof level. Enlarged views of the yellow square sections in **(A)** and **(C)** are shown in **(B)** and **(D)**, respectively. **(B, D)** TMT was defined as the length perpendicular to the longitudinal axis of the temporal muscle. The Sylvian fissure was used as a reference point in the anterior–posterior orientation. TMT, temporal muscle thickness.

We obtained brain MRI with commercial 1.5 or 3.0 T scanners, and the median time from stroke onset to MRI acquisition was 0.9 days (interquartile range, 0.4–2.2 days). Images included axial T2–weighted images and fluid-attenuated inversion recovery (FLAIR) images. Baseline TMT was measured on the axial plane of T2-weighted (parameters: echo time 97–130 ms; repetition time 4000–6600 ms; voxel size 1 × 1 × 3 to 7 mm^3^ and interslice gap 0–2.25 mm) or FLAIR images (parameters: echo time 76–160 ms; repetition time 6000–11 000 ms; voxel size 1 × 1 × 3 to 7 mm^3^ and interslice gap 0–2.25 mm), which were oriented parallel to the anterior commissure–posterior commissure line. We assessed TMT at the orbital roof level, taking measurements perpendicular to the longitudinal axis of the temporal muscle using the Picture Archiving and Communication System tool. The Sylvian fissure was defined as a reference point in the anterior–posterior orientation (Fig. 1).^17^ A stroke neurologist (D-SG) blinded to the clinical data measured the right side and the left side of the TMT, separately. The average value was then calculated for analysis. Intra-rater reliability was high, with an intraclass correlation coefficient of 0.90 (95% confidence interval [CI], 0.89–0.92). Considering the prevalence of sarcopenia among the stroke patient population,^3^ TMT was dichotomized a priori into low and high TMT using the 25th percentile as a cut-off point, and we defined low TMT as a surrogate of sarcopenia.

### Diffusion-weighted MRI registration and measurement

We obtained diffusion-weighted imaging (DWI) and quantified acute infarct volume on DWI using a previously validated and published protocol.^31,35^ Briefly, high signal intensity lesions on DWI were semi-automatically segmented and spatially normalized to the Montreal Neurological Institute templates using an in-house programme. Acute infarct volume on DWI was expressed as a percentage of total brain volume. As previously reported, inter-observer variability was minimal, and the intra-observer correlation coefficient for acute infarct volume ranged from 0.836 to 0.977.^31,35^

### Statistical analysis

Data are presented as mean ± standard deviation, median (interquartile range) and number (percentage), as appropriate. We compared baseline characteristics and outcomes between low and high TMT groups using Pearson’s chi-square test or Fisher’s exact test for categorical variables, Student’s *t*-test or the Mann–Whitney U-test for continuous variables, as appropriate. Missing data for infarct volume on DWI (*n* = 2) were replaced with the median of the entire population. To analyse the independent association between low TMT and outcomes including (i) dysphagia, (ii) END, (iii) in-hospital recovery, (iv) post-discharge recovery, and (v) chronic recovery, we performed multivariable logistic regression analysis. Predefined covariates that could potentially associate with outcomes after ischaemic stroke, including age, sex, admission NIHSS score, body mass index, pre-stroke mRS score, previous history of stroke, hypertension, diabetes, hyperlipidaemia, atrial fibrillation, coronary artery disease, smoking, stroke subtype, revascularization therapy, haemoglobin, total cholesterol and infarct volume were entered into the multivariable model.^31^ The associations of low TMT and functional outcomes including 3-months mRS score ≥3 and ≥4 as well as 1-year mRS score ≥ 3 and ≥4 were also analysed in the same manner. We also conducted mediation analysis, after adjusting for the same predefined covariates mentioned above, to evaluate the mediating roles of dysphagia, END, and post-stroke recovery at each period in the relationship between sarcopenia and poor functional outcomes.^36^ Possible mediator variables which showed independent association with sarcopenia and functional outcome were entered into the model.

Subgroup analyses were performed to investigate whether the association between low TMT and outcomes would differ according to initial presenting symptoms or AIS lesion locations. Presenting symptoms were classified with each NIHSS score item and lesion locations with cortex, corona radiata, basal ganglia/internal capsule, thalamus, midbrain, pons, medulla, and cerebellum. Multivariable logistic regression analysis with the same pre-defined covariates mentioned above and interaction terms for low TMT and each NIHSS score item or each lesion location, respectively, were used for these analyses. For rare event rates of subgroups, penalized logistic regression models were used. We also examined an effect modification low TMT has on post-stroke functional outcomes by infarct locations by using region of interest (ROI)-wise brain mapping after excluding two patients without DWI. Multivariable logistic regression for the brain mapping includes DWI lesion volume in each ROI, low TMT, interaction term for low TMT and DWI lesion volume, and the aforementioned covariates (except for total infarct volume because of the collinearity with DWI lesion volume). We then identified ROIs showing a significant interaction effect (*P*-for-interaction < 0.05). ROIs were drawn from, in combination, the Automated Anatomical Labelling (AAL) atlas,^37^ the Johns Hopkins University (JHU) white matter atlas^38^ and a brainstem atlas.^39^ Overlapping voxels between atlases were assigned priority in the order of the brainstem atlas, AAL atlas, and JHU atlas. Because we hypothesized that certain white matter tracts, including motor pathways, might critically affect outcomes of patients with sarcopenia, we conducted a pre-planned analysis that pooled left and right symmetric ROIs to overcome the small sample size.

For a sensitivity analysis, we examined the association between low TMT and an alternative definition of END (END_4_), an increase in NIHSS score of ≥4 points within 3 weeks after stroke onset.^40^ In addition, we queried the association of TMT, as a continuous variable, with outcomes by completing multivariable logistic regression analysis. Moreover, TMT was adjusted using the square of the height (TMT/height²) since inserting the square of height into the denominator minimizes the correlation between skeletal muscle mass and height across different sexes, age groups and study populations.^11^ TMT/height² was dichotomized into low and high TMT/height² using the 25th percentile of TMT/height² as a cut-off point in the same manner as described above. We then examined the associations TMT/height² has with outcomes. Data were analysed using STATA software 18.0 (STATA Corp., College Station, Texas, USA) and R version 4.0.5 (R Foundation for Statistical Computing). Brain mapping analysis was performed using MATLAB R2021b (Mathworks, Natick, MA, USA). Three-dimensional representation of ROIs was generated using the BrainNet Viewer.^41^ Statistical significance was considered at *P* < 0.05 and *P*-for-interaction < 0.05.

## Results

### Baseline characteristics

A total of 600 elderly patients were included in this study. Their mean age was 75.3 ± 6.1 years, and 50.5% were male (*n* = 303). The mean TMT was 6.7 ± 2.1 mm (right side, 6.8 ± 2.2 mm; left side, 6.7 ± 2.1 mm). The cut-off value for low TMT versus high TMT was 5.1 mm (Supplementary Fig. 1B). Compared to patients with high TMT, those with low TMT were more likely to be older; female; and have higher admission NIHSS score, lower body mass index, less frequent history of smoking, lower haemoglobin and albumin levels, and larger infarct volume (Table 1); when stratified by sex, the frequency of smoking did not differ significantly between the low and high TMT groups (Supplementary Table 2).

**Table 1:** Baseline characteristics: low vs. high TMT in patients with AIS^a^

	All (*n* = 600)	Low TMT group (*n* = 151)	High TMT group (*n* = 449)	*P*
Age, y	75.3 ± 6.1	78.2 ± 5.9	74.4 ± 5.9	<0.001
Male	303 (50.5)	38 (25.2)	265 (59.0)	<0.001
Height, cm	159.0 ± 9.2	154.8 ± 9.5	160.4 ± 8.7	<0.001
Weight, kg	60.6 ± 10.4	54.0 ± 10.1	62.8 ± 9.6	<0.001
Body mass index, kg/m^2^	23.9 ± 3.3	22.5 ± 3.4	24.4 ± 3.1	<0.001
TMT, mm	6.7 ± 2.1	4.0 ± 0.7	7.6 ± 1.6	<0.001
Admission NIHSS score	4 (2–6)	4 (2–8)	3 (2–6)	0.01
Pre-stroke mRS score				0.59
0	528 (88.0)	131 (86.8)	397 (88.4)	
1	72 (12.0)	20 (13.3)	52 (11.6)	
Previous stroke	119 (19.8)	26 (17.2)	93 (20.7)	0.35
Hypertension	496 (82.7)	125 (82.8)	371 (82.6)	0.97
Diabetes	268 (44.7)	67 (44.4)	201 (44.8)	0.93
Hyperlipidaemia	313 (52.2)	70 (46.4)	243 (54.1)	0.10
Smoking	261 (43.5)	40 (26.5)	221 (49.2)	<0.001
Atrial fibrillation	140 (23.3)	34 (22.5)	106 (23.6)	0.78
Coronary artery disease	57 (9.5)	14 (9.3)	43 (9.6)	0.91
Stroke subtype				0.92
Large artery atherosclerosis	211 (35.2)	54 (35.8)	157 (35.0)	
Small vessel occlusion	152 (25.3)	40 (26.5)	112 (24.9)	
Cardioembolism	110 (18.3)	26 (17.2)	84 (18.7)	
Undetermined	125 (20.8)	31 (20.5)	94 (20.9)	
Other determined	2 (0.3)	0 (0.0)	2 (0.5)	
Revasculization therapy	62 (10.3)	12 (8.0)	50 (11.1)	0.27
Haemoglobin, g/dL	13.4 ± 1.9	12.7 ± 1.8	13.7 ± 1.9	<0.001
Total cholesterol, mg/dL	170.3 ± 39.3	172.5 ± 43.1	169.6 ± 38.0	0.43
Total protein, g/dL	7.0 ± 0.6	6.9 ± 0.6	7.0 ± 0.6	0.24
Albumin, g/dL	4.2 ± 0.4	4.1 ± 0.4	4.3 ± 0.4	0.004
Infarct volume, % of brain	0.07 (0.02–0.42)	0.10 (0.03–0.57)	0.06 (0.02–0.37)	0.04

Data are presented as mean ± standard deviation, number (percentage), or median (interquartile range).

mRS, modified Rankin Scale; NIHSS, National Institutes of Health Stroke Scale; TMT, temporal muscle thickness.

^a^Patients were divided into low and high TMT groups by using the 25th percentile of TMT as the cut-off point.

### Low TMT associates with dysphagia, END, post-stroke recovery, and functional outcomes

Patients with low TMT (versus high TMT) had a higher incidence of dysphagia (36.4% versus 20.0%, *P* < 0.001) and experienced END more frequently (28.5% versus 14.0%, *P* < 0.001; Table 2). Notably, END due to stroke progression occurred more frequently in patients with low TMT than in those with high TMT. END by other mechanisms such as aspiration pneumonia and infection also tended to occur more frequently in the low TMT group, showing a marginal significance (*P* < 0.051). When END_4_—defined by the alternative criterion—was applied, its incidence was significantly higher in patients with low TMT compared to those with high TMT (12.6% versus 4.2%, *P* < 0.001). Moreover, patients with low TMT had less frequent in-hospital and post-discharge (up to 3 months) recovery than those with high TMT (33.3% versus 46.6%, *P* = 0.007; 22.3% versus 34.5%, *P* = 0.008, respectively). Although chronic recovery (from 3 months to 1 year) rates between the two groups did not significantly differ (24.2% versus 27.5%, *P* = 0.46; Table 2), patients with low TMT had poorer functional outcome at both 3 months (mRS score ≥ 3, 60.3% versus 33.4%, *P* < 0.001; mRS score ≥ 4, 37.1% versus 13.6%, *P* < 0.001) and 1 year (of 580 whose mRS scores at 1 year were available, mRS score ≥ 3, 55.7% versus 29.6%, *P* < 0.001; mRS score ≥ 4, 35.7% versus 15.2%, *P* < 0.001; Table 2).

**Table 2:** Clinical outcomes: low versus high TMT in patients with AIS^a^

	Low TMT group (*n* = 151)	High TMT group (*n* = 449)	*P*
Dysphagia	55 (36.4)	90 (20.0)	<0.001
END	43 (28.5)	63 (14.0)	<0.001
Stroke recurrence	1 (0.7)	2 (0.4)	>0.99
Stroke progression	33 (21.9)	50 (11.1)	0.001
Symptomatic haemorrhagic transformation	2 (1.3)	3 (0.7)	0.60
Others	3 (2.0)	1 (0.2)	0.051
Unknown	4 (2.6)	6 (1.3)	0.28
TIA	0 (0.0)	1 (0.2)	>0.99
In-hospital recovery^b^	46/138 (33.3)	191/410 (46.6)	0.007
Post-discharge recovery^c^	31/139 (22.3)	138/400 (34.5)	0.008
Chronic recovery^d^	30/124 (24.2)	101/367 (27.5)	0.46
3-mo mRS ≥3	91 (60.3)	150 (33.4)	<0.001
3-mo mRS ≥4	56 (37.1)	61 (13.6)	<0.001
1-y mRS ≥3^e^	78/140 (55.7)	130/440 (29.6)	<0.001
1-y mRS ≥4^e^	50/140 (35.7)	67/440 (15.2)	<0.001

Data are presented as number (percentage).

END, early neurological deterioration; mRS, modified Rankin Scale; NIHSS, National Institutes of Health Stroke Scale; TIA, transient ischaemic attack; TMT, temporal muscle thickness.

^a^Patients were divided into low and high TMT groups by using the 25th percentile of TMT as the cut-off point.

^b^Patients whose admission NIHSS was 0 (*n* = 52) were excluded from the analysis.

^c^Patients whose discharge mRS score was 0 (*n* = 56) or 6 (*n* = 5) were excluded from the analysis.

^d^Patients whose 3-month mRS score was 0 (*n* = 69) or 6 (*n* = 21) were excluded from the analysis. Of these patients, those who were lost to follow-up at 1 year after the index stroke (*n* = 19) were also excluded from the analysis.

^e^Patients lost to follow-up at 1 year after the index stroke (*n* = 20) were excluded from the analysis.

In logistic regression analyses (Table 3), low TMT was independently associated with dysphagia (adjusted odds ratio [aOR] 1.89, 95% CI 1.06–3.37, *P* = 0.03) and END (aOR 2.75, 95% CI 1.61–4.71, *P* < 0.001), as well as with END_4_ based on the alternative definition (aOR 2.36, 95% CI 1.05–5.31, *P* = 0.04). Low TMT was also associated with in-hospital recovery (OR 0.59, 95% CI 0.38–0.86, *P* = 0.007), but this association was only marginally significant when adjusted for covariates (aOR 0.67, 95% CI 0.42–1.06, *P* = 0.08). Low TMT also showed an independent association with post-discharge (up to 3 months) recovery (aOR 0.56, 95% CI 0.34–0.94, *P* = 0.03). Although there was no significant independent association between low TMT and chronic (3 months to 1 year) recovery (aOR 1.13, 95% CI 0.66–1.94, *P* = 0.66; Table 3), low TMT was independently associated with poor functional outcome at both 3 months and 1 year (all *P* < 0.05; Table 3).

**Table 3:** Logistic regression analyses to assess the relationship between low TMT and outcomes in patients with AIS^a^

	Univariable analysis : OR (95% CI)	*P*	Multivariable analysis : OR (95% CI)^b^	*P*
Dysphagia	2.29 (1.53–3.42)	<0.001	1.89 (1.06–3.37)	0.03
END	2.44 (1.57–3.80)	<0.001	2.75 (1.61–4.71)	<0.001
In-hospital recovery^c^	0.57 (0.38–0.86)	0.007	0.67 (0.42–1.06)	0.08
Post-discharge recovery^d^	0.54 (0.35–0.85)	0.008	0.56 (0.34–0.94)	0.03
Chronic recovery^e^	0.84 (0.53–1.35)	0.47	1.13 (0.66–1.94)	0.66
3-mo mRS ≥ 3	3.02 (2.07–4.42)	<0.001	2.89 (1.70–4.92)	<0.001
3-mo mRS ≥ 4	3.75 (2.45–5.74)	<0.001	4.01 (2.12–7.59)	<0.001
1-y mRS ≥ 3^f^	3.00 (2.03–4.44)	<0.001	2.50 (1.48–4.25)	0.001
1-y mRS ≥ 4^f^	3.09 (2.01–4.77)	<0.001	2.17 (1.17–4.02)	0.01

CI, confidence interval; END, early neurological deterioration; mRS, modified Rankin Scale; NIHSS, National Institutes of Health Stroke Scale; OR, odds ratio; TMT, temporal muscle thickness.

^a^Low TMT was defined as below the 25th percentile of TMT.

^b^Data were adjusted for age, sex, admission NIHSS, body mass index, pre-stroke mRS, previous history of stroke, hypertension, diabetes, hyperlipidaemia, smoking, atrial fibrillation, coronary artery disease, stroke subtype, revascularisation therapy, total cholesterol, haemoglobin and infarct volume.

^c^Patients whose admission NIHSS was 0 (*n* = 52) were excluded from the analysis.

^d^Patients whose discharge mRS score was 0 (*n* = 56) or 6 (*n* = 5) were excluded from the analysis.

^e^Patients whose 3-month mRS score was 0 (*n* = 69) or 6 (*n* = 21) were excluded from the analysis. Of these patients, those who were lost to follow-up at 1 year after the index stroke (*n* = 19) were also excluded from the analysis.

^f^Patients lost to follow-up at 1 year after the index stroke (*n* = 20) were excluded from the analysis.

Mediation analysis (Supplementary Fig. 2) showed that dysphagia, END, and post-discharge recovery significantly mediated the association sarcopenia has with poor functional outcome (mRS ≥ 3) at 3 months, accounting for 45.3% of total effect. Sarcopenia also affected long-term (1-year) functional outcome, either directly or through END.

### Low TMT has greater associations with outcomes in patients with motor symptoms or pontine lesions

**Figure 2:**
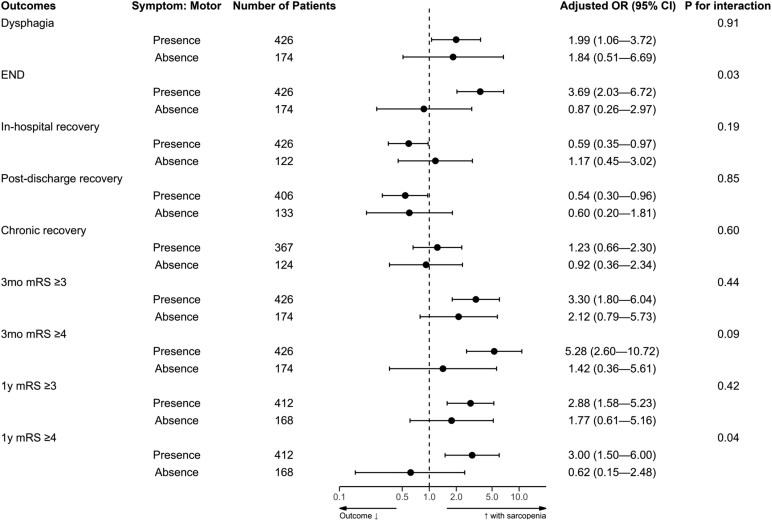
**Low TMT associates with stroke outcomes in elderly AIS patients with versus without motor deficits.** Multivariable logistic regression analyses were performed, including an interaction term between low TMT and the presence (versus absence) of motor deficits, defined as a motor score ≥ 1 on NIHSS score items 5a–6b. AIS, acute ischaemic stroke; CI, confidence interval; END, early neurological deterioration; mRS, modified Rankin Scale; NIHSS, National Institutes of Health Stroke Scale; OR, odds ratio.

**Figure 3:**
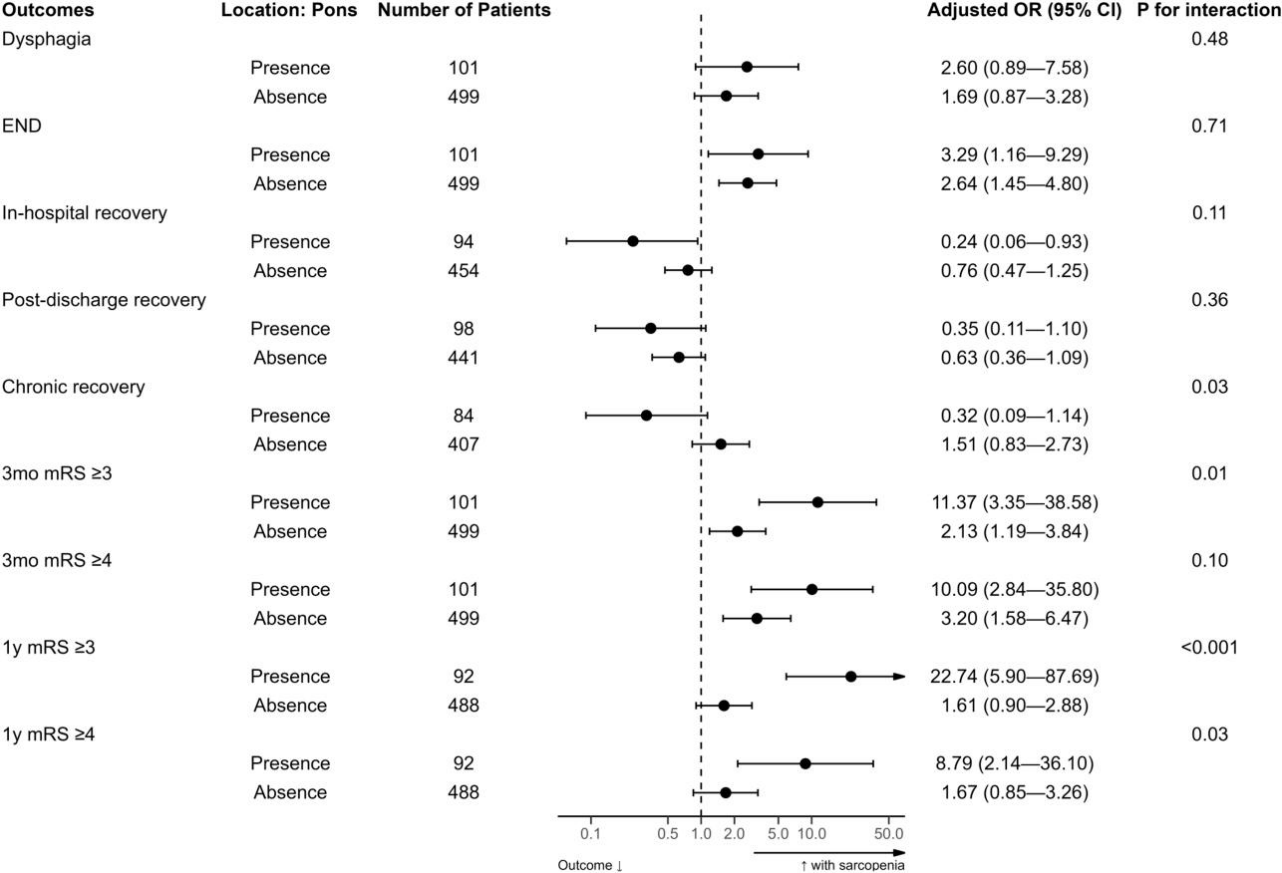
**Low TMT associates with stroke outcomes in elderly AIS patients with versus without pontine lesions.** Multivariable logistic regression analyses were performed, with an interaction term between low TMT and the presence (versus absence) of pontine lesions. AIS, acute ischaemic stroke; CI, confidence interval; END, early neurological deterioration; mRS, modified Rankin Scale; OR, odds ratio.

To identify specific subgroups where sarcopenia had a stronger effect on post-stroke outcomes, we analysed the interaction between low TMT and initial symptoms. In patients with motor deficit, compared to those without, low TMT had a greater effect on END and 1-year mRS score ≥ 4 (*P*-for-interaction = 0.03 and 0.04, respectively; Fig. 2). In patients with (versus without) facial palsy, low TMT had a greater effect on 1-year mRS score ≥ 4 (*P*-for-interaction = 0.03; Supplementary Fig. 3). Low TMT also showed a greater effect on 1-year mRS score ≥ 3 in patients with (versus without) dysarthria (*P*-for-interaction = 0.02; Supplementary Fig. 4). The other (non-motor) symptoms showed no significant interaction effects (Supplementary Figs 5–11). In patients with pontine (versus non-pontine) lesions, low TMT had greater effects on chronic recovery, 3-month mRS score (≥3), and 1-year mRS score (≥3 and ≥4), with *P*-for-interactions of 0.03, 0.01, <0.001, and 0.03, respectively (Fig. 3). Regarding other lesion locations, no significant interactions were observed (Supplementary Figs 12–18).

### Brain mapping to show low TMT has greater effects on outcomes after motor pathway infarctions

**Figure 4:**
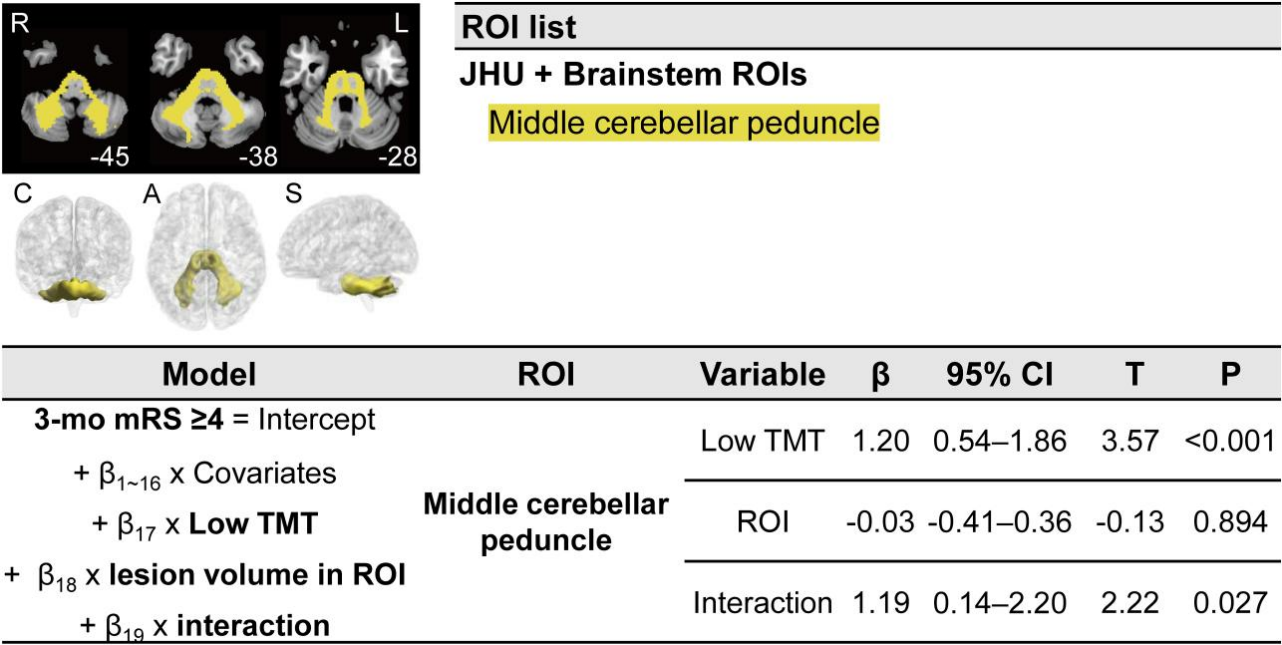
**ROI-based multivariable brain mapping and regression analyses to show infarct location-dependent associations between low TMT and poor functional outcomes following AIS.** ROI-based analysis showing a brain region in which low TMT has a greater effect on 3-month functional outcomes. Top left: L and R denote left and right, respectively. Middle left: a three-dimensional representation of the significant ROIs. C, A and S denote coronal, axial and sagittal views, respectively. Top right: a complete list of significant ROIs, with labels from the AAL, JHU, and Brainstem atlases. Bottom: results of multivariable regression analyses (*n* = 598). In patients with lesions involving middle cerebellar peduncle, compared to those without, low TMT had a greater impact on poor 3-month functional outcome (mRS score ≥4). *β*s indicate *β* coefficients, and *T*s indicates *t* statistics for independent variables in the regression models. AAL, automated anatomical labelling; CI, confidence interval; JHU, Johns Hopkins University; mRS, modified Rankin Scale; ROI, region of interest; TMT, temporal muscle thickness.

**Figure 5:**
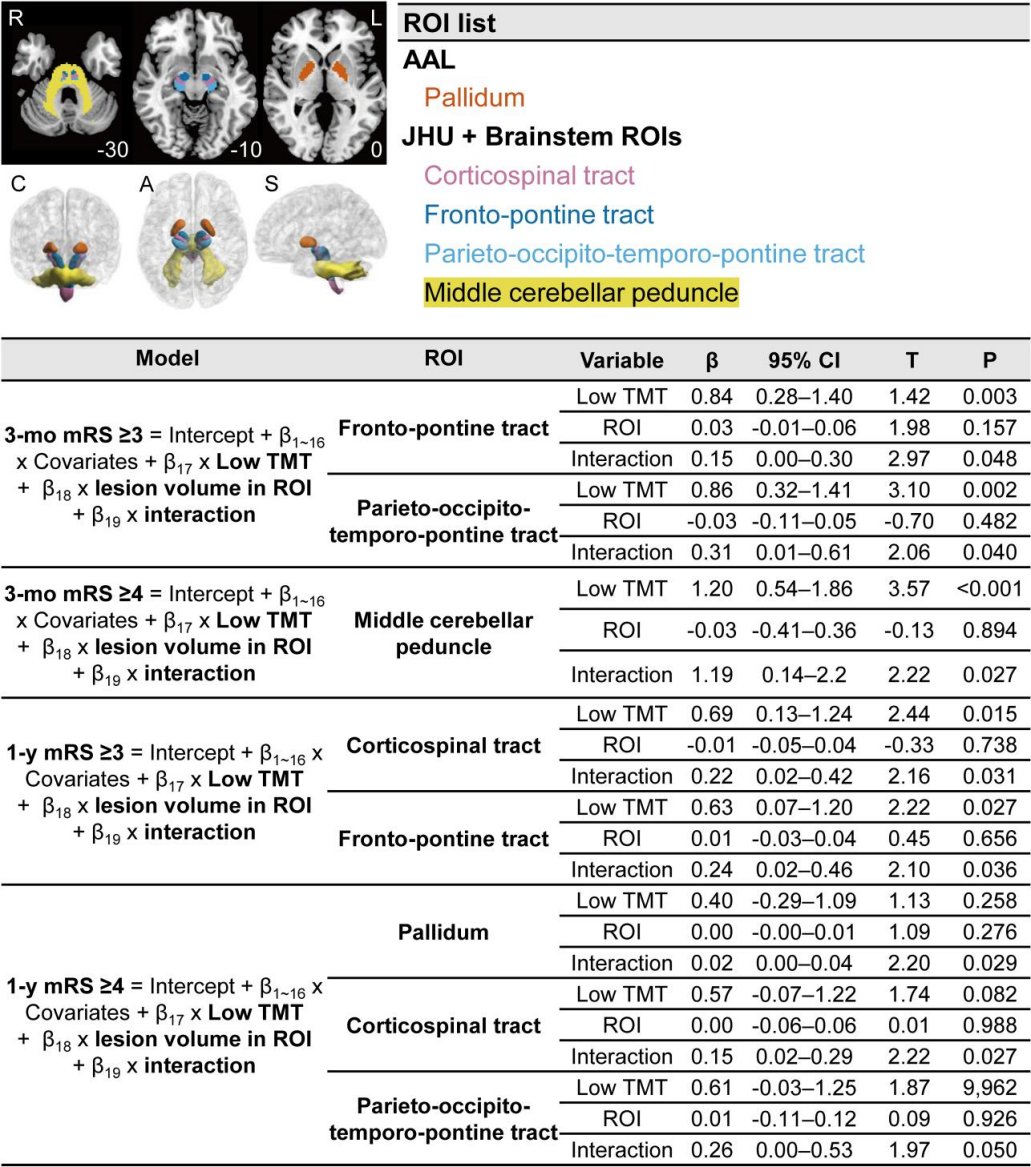
**Bilateral pooled ROI-based multivariable brain mapping and regression analyses to show infarct location-dependent associations between low TMT and poor functional outcomes following AIS.** Pooled analysis of symmetric ROIs from both hemispheres, showing significant brain regions where low TMT has a greater effect on 3-month and 1-year functional outcomes. Top left: L and R denote left and right, respectively. Middle left: a three-dimensional representation of the significant ROIs. C, A and S denote coronal, axial and sagittal views, respectively. Top right: a complete list of significant ROIs, with labels from the AAL, JHU, and Brainstem atlases. Bottom: results of multivariable regression analyses (*n* = 598). *β*s indicate *β* coefficients, and Ts indicates *t* statistics for independent variables in the regression models. AAL, automated anatomical labelling; CI, confidence interval; JHU, Johns Hopkins University; mRS, modified Rankin Scale; ROI, region of interest; TMT, temporal muscle thickness.

ROI-wise brain mapping analysis revealed that one ROI showed interactions with low TMT (middle cerebellar peduncle for mRS score ≥4 at 3 months; Fig. 4). When we performed pooled analysis combining symmetric ROIs from both the left and right hemispheres, we found that multiple ROIs showed interactions with low TMT on functional outcomes: fronto-pontine tract and parieto-occipito-temporo-pontine tract for mRS score ≥3 at 3 months; middle cerebellar peduncle for mRS score ≥4 at 3 months; corticospinal tract and fronto-pontine tract for mRS score ≥3 at 1 year; pallidum, corticospinal tract, and parieto-occipito-temporo-pontine tract for mRS score ≥4 at 1 year (Fig. 5).

### Sensitivity analyses

Sensitivity analyses were conducted using TMT as a continuous variable. The decreased TMT showed independent associations with dysphagia (aOR [per 1 mm decrease] 1.23, 95% CI 1.07–1.42, *P* = 0.003), END (aOR [per 1 mm decrease] 1.25, 95% CI 1.09–1.42, *P* = 0.001), and in-hospital recovery (aOR [per 1 mm decrease] 0.81, 95% CI 0.73–0.90, *P* < 0.001) but not with post-discharge and chronic recovery (aOR [per 1 mm decrease] 0.96, 95% CI 0.86–1.07, *P* = 0.47 and aOR [per 1 mm decrease] 1.06, 95% CI 0.94–1.19, *P* = 0.37, respectively; Supplementary Table 3). Reduced TMT was significantly associated with poor functional outcomes at 3 months and 1 year (all *P* < 0.05; Supplementary Table 3).

The mean TMT/height² was 2.7 ± 0.8 mm/m^2^. It was dichotomized at 2.0 mm/m^2^ for low versus high TMT/height^2^. In patients with low TMT/height², 88.7% (133/150) belonged to the low-TMT group, while in those with high TMT/height², 96.0% (432/450) belonged to the high-TMT group. Patients with low TMT/height^2^ were more likely to experience dysphagia (38.0% versus 19.6%, *P* < 0.001) and END (27.3% versus 14.4%, *P* < 0.001) but had a lower likelihood of in-hospital recovery (35.3% versus 46.0%, *P* = 0.03). However, post-discharge recovery and chronic recovery rates did not differ significantly between the low- and high-TMT/height^2^ groups: 26.6% versus 33.0% (*P* = 0.16) and 25.2% versus 27.2% (*P* = 0.67), respectively (Supplementary Table 4). Patients with low TMT/height^2^ were more likely to have poor functional outcomes at 3 months and 1 year than those with high TMT/height^2^ (all *P* < 0.001; Supplementary Table 4). Lastly, low TMT/height^2^ showed significant independent associations with dysphagia (aOR 2.12, 95% CI 1.23–3.65, *P* = 0.007) and END (aOR 2.36, 95% CI 1.41–3.93, *P* = 0.001) but not with post-stroke recovery at any of the three periods (all adjusted *P* > 0.05; Supplementary Table 5). Low TMT/height^2^ showed significant independent associations with poor functional outcomes at 3 months and at 1 year (Supplementary Table 5).

## Discussion

This study found that patients with sarcopenia, as indicated by low TMT, had a ∼2-fold higher rate of dysphagia, ∼3-fold higher rate of END, and a ∼2-fold lower rate of post-discharge recovery, which significantly contributed to poor functional outcomes after stroke, according to the mediation analysis. In addition, statistical and brain mapping analyses showed that effects of sarcopenia on post-stroke outcomes were greater in patients with motor deficits, bulbar symptoms, or motor pathway-related lesions.

**Figure 6:**
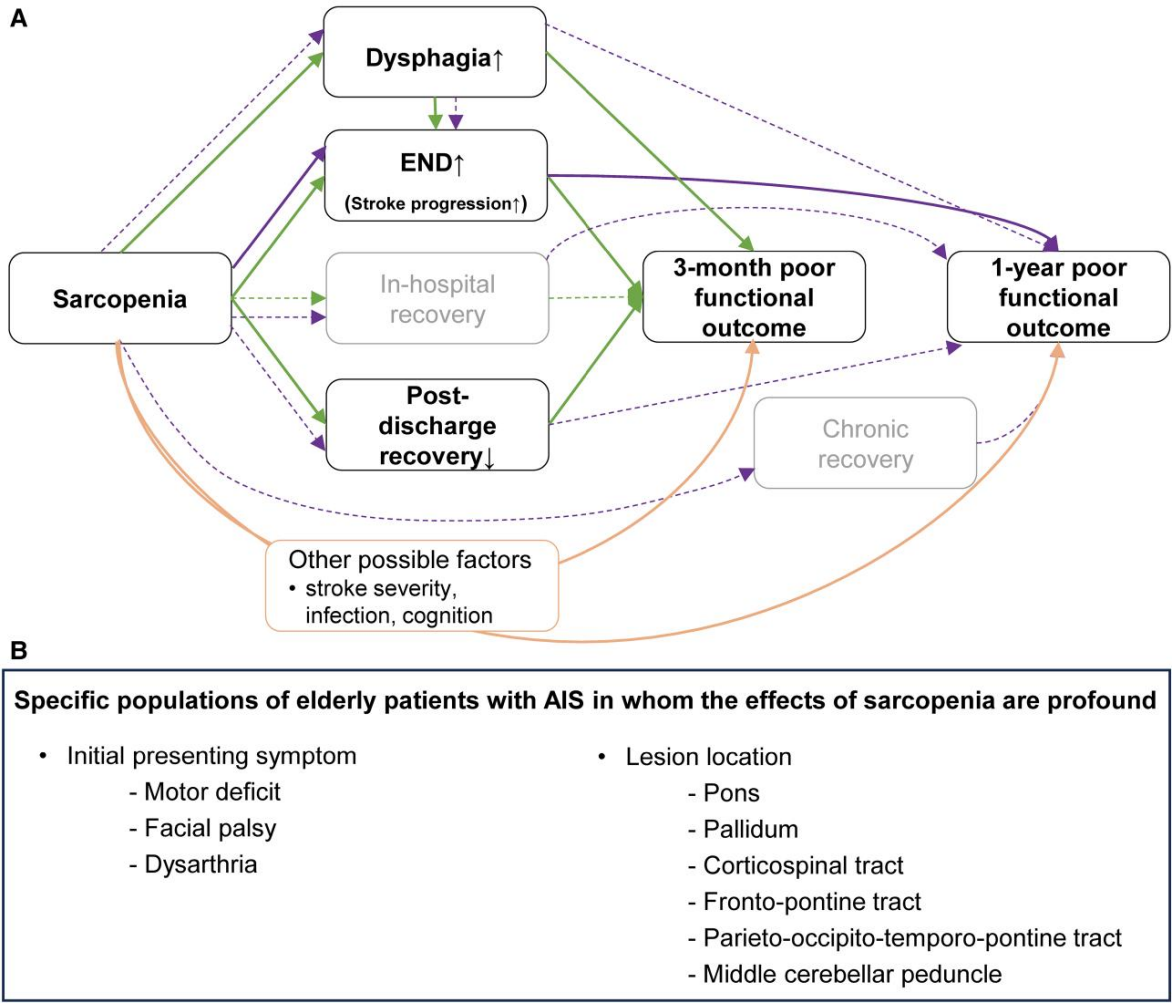
**Graphical representation of proposed mechanisms by which sarcopenia affects functional outcomes following AIS.** (**A**) Diagram based on our mediation analysis results and the literature on other factors^6,18,20^ which may potentially link sarcopenia to functional outcomes (note that the literature has only shown associations between sarcopenia and other factors, not the mediating roles of factors in long-term functional outcomes). Solid and dashed arrows indicate significant and insignificant mediators, respectively. (**B**) Specific populations of elderly AIS patients who are likely to be more strongly affected by sarcopenia. AIS, acute ischaemic stroke; END, early neurological deterioration.

The mechanisms underlying the association between sarcopenia and poor functional outcomes in elderly patients with AIS are likely multifactorial (Fig. 6). First, we found that patients with sarcopenia were more likely to experience END, a well-known prognostic factor.^42^ Compared to a previous study regarding sarcopenia and END,^19^ we explored END causes in more detail. Individuals with low TMT had stroke progression more often than those with high TMT. Since patients with sarcopenia have reduced physical and functional reserves, they might be more susceptible to neurological aggravation mediated by infarct growth. Other END causes, such as systemic infection and aspiration pneumonia, also tended to be more frequent in the low (versus high) TMT group. Sarcopenia can involve swallowing-related muscles, leading to dysphagia and increasing the risk of aspiration pneumonia.^16-18^ We found that dysphagia mediates the effect sarcopenia exerts on poor functional outcomes at 3 months, either directly or through END. This impact may be partly attributed to the association of dysphagia with malnutrition and aspiration pneumonia.^43^ It was impossible to investigate independent associations between low TMT and every recorded cause of END, given the low number of events. Further inquiry with a larger sample size is required.

As a second mechanism, we found that sarcopenia impaired post-stroke recovery during a specific period, with all differences in recovery becoming apparent by 3 months and no new differences emerging between 3 months and 1 year. Recovery mechanisms following ischaemic stroke vary by time period.^33,34^ Reperfusion and neuroprotection, which typically span days or a few weeks, are crucial for in-hospital recovery.^32-34,44^ Modulation of inflammation and reversal of diaschisis, a decrease in neuronal activity in connected brain areas due to disrupted functional connectivity, are key for post-discharge recovery over several months.^33,34^ For later chronic recovery, it is important for neural networks, which encompass cognitive domains such as memory, attention, executive functions, language, and visuospatial abilities, to be repaired or reconfigured.^33,34^ Aligning with distinct recovery mechanisms that operate during different periods after stroke,^33,34^ low TMT was independently associated with post-discharge recovery but not linked to in-hospital or chronic recovery. Although low TMT was associated with in-hospital recovery, this association was only marginally significant after adjusting for covariates including revascularization therapy. The inflammation-related post-discharge recovery process could be hindered by sarcopenia, which is often accompanied by a systemic pro-inflammatory state^45^ that can also affect central nervous system diaschisis by exacerbating neuronal dysfunction^34^ and chronic low-grade inflammation within muscles.^45^ A compromised nervous system with a decline in motor neurons and neuromuscular junctions in sarcopenia^46^ may impair post-discharge motor recovery.^47^ Sarcopenia may also reduce cognitive reserve and hippocampal function, possibly due in part to decreased physical activity and lower brain-derived neurotrophic factor levels.^48,49^ However, during the chronic recovery period, when cognitive domains are important targets,^33,34^ sarcopenia did not significantly affect post-stroke recovery. Further investigation is required.

A third mechanistic effect of sarcopenia is more pronounced in specific AIS populations. Patients with motor deficits, as opposed to non-motor deficits, are more affected by the presence of sarcopenia, which is often accompanied by reduced mobility and increased risk of falls and fracture.^26^ In addition, acute stroke-mediated facial palsy and dysarthria combined with chronic sarcopenia-related bulbar muscle atrophy^17^ can elevate the incidence of malnutrition^43^ and pneumonia,^18^ thereby worsening long-term functional outcomes, as our study demonstrates. Regarding lesion locations, sarcopenia had a stronger impact on 3-month and 1-year functional outcomes in patients with pontine infarcts, which often manifest with both limb weakness and bulbar symptoms.^50^ Moreover, sarcopenia impeded chronic recovery in patients with pontine lesions, possibly due, at least in part, to pons’ extensive functional connections to other brain regions^51^ and documented decline in supraspinal motor drive during sarcopenia.^46^ In support, post-stroke motor recovery has been shown to rely heavily on the ponto-medullary reticulospinal pathway,^52^ and reduced reticulospinal output has been observed in older individuals with sarcopenia.^53^

Supporting the statistical findings presented above, the ROI-wise brain mapping analysis showed that sarcopenia had a greater impact on functional outcomes in patients with infarctions in brain regions—namely the pallidum, fronto-pontine tract, parieto-occipito-temporo-pontine tract, and middle cerebellar peduncle—that are responsible for motor planning, execution, and control,^39,54^ as well as the corticospinal tract, which is involved in motor strength.

This study has several limitations. First, as an observational study, it may be subject to unmeasured confounders, such as socioeconomic status, marital status, living situation, cognitive function, dietary habits and physical activity. Second, TMT primarily reflect muscle quantity rather than quality, although prior studies have shown that TMT correlates with hand grip strength, which may serve as a proxy for muscle quality.^23^ Third, the lack of serial TMT assessments after discharge limits our understanding of sarcopenia progression during the post-discharge period. Fourth, we assessed the presence or absence of dysphagia, rather than its severity. Fifth, the mRS score, which is used to define chronic recovery, may not sensitively reflect cognitive or language functions.^55^ Sixth, not applying multiple comparisons correction carries a potential risk of false positives, which we acknowledge as a study limitation, although interaction analyses may be interpreted with less stringent criteria. Seventh, data from a single centre involving a single-ethnicity population might have limited generalisability.

In conclusion, sarcopenia negatively affected post-stroke functional outcomes in elderly AIS patients by increasing the risk of dysphagia and END and reducing the likelihood of post-discharge recovery. Further, sarcopenia showed more pronounced effects in patients with motor deficits or bulbar symptoms and in those with lesions involving pons and motor tracts related to motor strength as well as motor planning, execution, and control. Understanding and identifying the mechanisms underlying sarcopenia-related effects on functional outcomes can inform comprehensive, time-specific approaches to personalized management for elderly AIS patients. Our results suggest that physicians should pay attention to sarcopenia and consider routinely measuring TMT by using baseline brain imaging.

## Supplementary Material

fcaf386_Supplementary_Data

## Data Availability

The data that support the findings of this study are available from the corresponding author upon reasonable request. The codes generated and used in this work are publicly available at GitHub (https://github.com/dongsk002/TMT-and-stroke).
