# Supplementary material for: Mechanisms of how sarcopenia affects functional outcomes in acute ischaemic stroke
Source: Brain Commun. 2025 Nov 11;7(6):fcaf386. doi: 10.1093/braincomms/fcaf386 (PMC12603365; doi:10.1093/braincomms/fcaf386)
Supplement: fcaf386_Supplementary_Data [file fcaf386_supplementary_data.pdf]

Supplementary Materials

Mechanisms of How Sarcopenia Affects Functional Outcomes in Acute Ischaemic Stroke

Supplementary Tables

Supplementary Table 1. Summary of the Literature on Associations of Prestroke Sarcopenia or Muscle Mass Deficits with Stroke Outcomes, Compared to the Present Study

Supplementary Table 2. Sex-stratified Comparison of Smoking Frequency Between Low and High Temporal Muscle Thickness (TMT) in Patients with Acute Ischaemic Stroke

Supplementary Table 3. Sensitivity Analyses: Logistic Regression Analyses to Assess Temporal Muscle Thickness as a Continuous Variable in Relation to Outcomes in Patients with Acute Ischaemic Stroke

Supplementary Table 4. Sensitivity Analyses: Clinical Outcomes by Temporal Muscle Thickness / Height^2 in Patients with Acute Ischaemic Stroke

Supplementary Table 5 Sensitivity Analyses: Logistic Regression Analyses to Assess the Relationships between Low Temporal Muscle Thickness / Height^2 and Outcomes in Patients with Acute Ischaemic Stroke

Supplementary Figures

Supplementary Figure 1. (A) Study flowchart and (B) histogram of temporal muscle thickness.

Supplementary Figure 2. Mediation analysis for pathways from sarcopenia to poor functional outcomes (modified Rankin Scale ≥3) at 3 months and 1 year.

Supplementary Figure 3. Associations between low temporal muscle thickness and stroke outcomes in elderly acute ischaemic stroke patients with vs. without facial palsy.

Supplementary Figure 4. Associations between low temporal muscle thickness and stroke outcomes in elderly acute ischaemic stroke patients with vs. without dysarthria.

Supplementary Figure 5. Associations between low temporal muscle thickness and stroke outcomes in elderly acute ischaemic stroke patients with vs. without loss of consciousness.

Supplementary Figure 6. Associations between low temporal muscle thickness and stroke outcomes in elderly acute ischaemic stroke patients with vs. without gaze abnormalities.

Supplementary Figure 7. Associations between low temporal muscle thickness and stroke outcomes in elderly acute ischaemic stroke patients with vs. without abnormalities in either visual acuity or visual field.

Supplementary Figure 8. Associations between low temporal muscle thickness and stroke outcomes in elderly acute ischaemic stroke patients with vs. without ataxia.

Supplementary Figure 9. Associations between low temporal muscle thickness and stroke outcomes in elderly acute ischaemic stroke patients with vs. without sensory abnormalities.

Supplementary Figure 10. Associations between low temporal muscle thickness and stroke outcomes in elderly acute ischaemic stroke patients with vs. without aphasia.

Supplementary Figure 11. Associations between low temporal muscle thickness and stroke outcomes in elderly acute ischaemic stroke patients with vs. without extinction and inattention.

Supplementary Figure 12. Associations between low temporal muscle thickness and stroke outcomes in elderly acute ischaemic stroke patients with vs. without cortical lesions.

Supplementary Figure 13. Associations between low temporal muscle thickness and stroke outcomes in elderly acute ischaemic stroke patients with vs. without corona radiata lesions.

Supplementary Figure 14. Associations between low temporal muscle thickness and stroke outcomes in elderly acute ischaemic stroke patients with vs. without basal ganglia or internal capsule lesions.

Supplementary Figure 15. Associations between low temporal muscle thickness and stroke outcomes in elderly acute ischaemic stroke patients with vs. without thalamic lesions.

Supplementary Figure 16. Associations between low temporal muscle thickness and stroke outcomes in elderly acute ischaemic stroke patients with vs. without midbrain lesions.

Supplementary Figure 17. Associations between low temporal muscle thickness and stroke outcomes in elderly acute ischaemic stroke patients with vs. without medullary lesions.

Supplementary Figure 18. Associations between low temporal muscle thickness and stroke outcomes in elderly acute ischaemic stroke patients with vs. without cerebellar lesions.

Supplementary References

Supplementary Table 1. Summary of the Literature on Associations of Prestroke Sarcopenia or Muscle Mass Deficits with Stroke Outcomes, Compared to the Present Study

Year	Author	Population	No. of subjects	Mean/ median age, y[a]	Diagnosis of sarcopenia / muscle mass deficit	Association of sarcopenia/muscle mass deficit with stroke outcomes	Exclusion rate[b]	Mechanism[c]
**Studies regarding the association of sarcopenia/muscle mass deficit with functional outcome**								
2020	Ohyama K et al.[S1]	AIS ≥65	164	77.3	ASMI (by BIA)	Muscle mass deficit was associated with mRS ≥3 at discharge.	Not reported	NA
2020	Abe T et al.[S2]	AIS	107	76.0	ASMI (by BIA)	Muscle mass deficit was associated with mRS ≥4 at discharge.	21.9% (70/320)	NA
2024	Han M et al.[S3]	AIS	660	65.6	ASMI (by BIA)	Muscle mass deficit was associated with initial stroke severity.<br>Muscle mass deficit was associated with mRS ≥2 at discharge.	70.5% (1895/2687)	Sarcopenia affects discharge functional outcome mediated by initial stroke severity.
2022	Lee H et al.[S4]	Mild AIS / TIA	568	65.5	ASMI (by BIA) and muscle strength (by MRC score)	Sarcopenia (both muscle mass deficit and low muscle strength) was associated with mRS ≥2 at 3 months, more evident in cases of low muscle mass in the lower extremities.	32.2% (755/2346)	The impact of sarcopenia on functional outcome is profound in specific population: those with low muscle mass in the lower extremities.
2023	Honma K et al.[S5]	AIS	189	74.8	ASMI (by BIA)	ASMI was associated with mRS ≥3 at discharge.	1.4% (3/210)	NA
2022	Nozoe M et al.[S6]	AIS and haemorrhagic stroke ≥65	317 (AIS: 262)	76	SARC-F	Sarcopenia risk was associated with mRS ≥4 at 3 months.	26.2% (162/619)	NA
2019	Nozoe M et al.[S7]	AIS and haemorrhagic stroke >65	152 (AIS: 123)	76	SARC-F	Sarcopenia risk was associated with mRS ≥4 at 3 months.	25.8% (62/240)	NA

2023	Lee SH et al.[S8]	AIS	653	65.9	ASMI (by DEXA)	ASMI was associated with mRS ≥4, not mortality, at 3 months.<br><br>Compared to the groups with the highest ASMI quartile, the other three groups with lower quartiles did not show independent associations with mRS ≥4 at 3 months.<br><br>Sarcopenia (both muscle mass deficit and low muscle strength) was not associated with mRS ≥4 and mortality at 3 months.	49.4% (870/1760)	NA
2023	Lin YH et al.[S9]	AIS with EVT	657	72.0	TMT (by CTA)	TMT was associated with mRS ≥3 at 3 months, especially in older adults.	0.9% (6/677)	NA
2021	Nozoe M et al.[S10]	AIS and haemorrhagic stroke ≥65	289 (AIS 234)	76	TMT (by CT), SARC-F	TMT was associated with sarcopenia risk.<br><br>TMT was not associated with mRS ≥4 at 3 months.	TMT: 9.5% (61/643), SARC-F: 26.6% (171/643)	NA
2023	Tutal Gursoy G et al.[S11]	AIS	147	67.6	TMT (by CT)	TMT did not show independent associations with mRS ≥3 or mortality at 3 months.	Not reported	NA
**Studies regarding the association of sarcopenia/muscle mass deficit with stroke outcomes other than functional outcome**								
2019	Nozoe M et al.[S12]	AIS and haemorrhagic stroke ≥65	183 (AIS 150)	75	SARC-F	Sarcopenia risk was associated with initial stroke severity.	22.8% (72/316)	NA
2023	Fukuma K et al.[S13]	AIS and haemorrhagic stroke ≥60	350 (AIS 270)	77	Calf circumference, grip strength, and ASMI (by BIA)	Sarcopenia was associated with dysphagia and aspiration pneumonia.	Not reported	NA

2021	Sakai K et al.[S14]	AIS and haemorrhagic stroke	70 (AIS 56)	75.6	TMT (by T2 MRI)	TMT was associated with severity of dysphagia at discharge.	54.5% (84/154)	NA
2023	Song X et al.[S15]	AIS ≥60	1002	72.0	SARC-F	Sarcopenia risk was independently associated with stroke-associated infection.	5.7% (65/1133)	NA
2023	Imamura M et al.[S16]	AIS ≥65	290	76	SARC-F	Sarcopenia risk was associated with END.	28.5% (166/583)	NA
2023	Namgung HG et al.[S17]	AIS ≥65	126	79	TMT (by T2 MRI), ASMI (by BIA)	TMT was associated with early cognitive impairment (within 2 weeks of stroke onset).<br>TMT was correlated with ASMI.	Not reported	NA
2022	Li YX et al.[S18]	AIS	265	67.4	TMT (by T1 MRI)	Muscle mass deficit (TMT below its median value) was associated with mortality.	Not reported	NA
2024	Gwak DS et al. (present study)	AIS ≥65	600	75.3	TMT (by T2 or FLAIR MRI)	Muscle mass deficit (TMT below the 25th percentile) was associated with dysphagia, END, and post-discharge recovery.<br>Muscle mass deficit was associated with mRS (≥3 and ≥4) at 3 months and 1 year.	1.1% (9/822)	Sarcopenia affects poststroke functional outcome via dysphagia, END, and post-discharge recovery.<br>The impact of sarcopenia on functional outcomes is profound in specific populations with motor deficits, bulbar symptoms, or lesions in pons or motor tracts.

[a]The underscore indicates the median age

[b]Percentage of screened patients excluded from the study due to failure to assess sarcopenia or muscle mass deficit.

[c]NA indicates that the study did not investigate the mechanism by which sarcopenia affects poststroke functional outcome.

AIS, acute ischaemic stroke; ASMI, appendicular skeletal muscle mass index; BIA, Bioelectrical impedance analysis; CTA, computed tomography angiography; DEXA,

dual-energy X-ray absorptiometry; END, early neurological deterioration; EVT, endovascular thrombectomy; FLAIR, fluid-attenuated inversion recovery; MRC, medical research council; mRS, modified Rankin Scale; NA, not applicable; SARC-F, Strength, assistance with walking, rising from a chair, climbing stairs, and falls; TIA, transient ischaemic attack; TMT, temporal muscle thickness.

Supplementary Table 2. Sex-stratified Comparison of Smoking Frequency Between Low and High Temporal Muscle Thickness (TMT) in Patients with Acute Ischaemic Stroke[a]

	Male			Female		
	Low TMT Group (*n* = 38)	High TMT Group (*n* = 265)	*P*	Low TMT Group (*n* = 113)	High TMT Group (*n* = 184)	*P*
Smoking	31 (81.6)	210 (79.2)	0.74	9 (8.0)	11 (6.0)	0.51

Data are presented as number (percentage).

[a]Patients were divided into low and high TMT groups by using the 25th percentile of TMT as the cut-off point.

Supplementary Table 3. Sensitivity Analyses: Logistic Regression Analyses to Assess TMT as a Continuous Variable in Relation to Outcomes in Patients with Acute Ischaemic Stroke[a]

	Univariable analysis : OR (95% CI)	*P*	Multivariable analysis : OR (95% CI)[b]	*P*
Dysphagia	1.24 (1.13-1.36)	<0.001	1.23 (1.07-1.42)	0.003
END	1.21 (1.09-1.34)	<0.001	1.25 (1.09-1.42)	0.001
In-hospital recovery[c]	0.82 (0.75-0.89)	<0.001	0.81 (0.73-0.90)	<0.001
Post-discharge recovery[d]	0.94 (0.86-1.03)	0.19	0.96 (0.86-1.07)	0.47
Chronic recovery[e]	0.98 (0.88-1.08)	0.62	1.06 (0.94-1.19)	0.37
3-mo mRS ≥3	1.33 (1.22-1.45)	<0.001	1.39 (1.22-1.58)	<0.001
3-mo mRS ≥4	1.34 (1.20-1.49)	<0.001	1.35 (1.15-1.59)	<0.001
1-y mRS ≥3[f]	1.32 (1.20-1.44)	<0.001	1.32 (1.16-1.49)	<0.001
1-y mRS ≥4[f]	1.28 (1.15-1.43)	<0.001	1.21 (1.04-1.40)	0.02

[a]The OR values for each 1 mm decrease in TMT are presented.

[b]Data were adjusted for age, sex, admission NIHSS, body mass index, pre-stroke mRS, previous history of stroke, hypertension, diabetes, hyperlipidaemia, smoking, atrial fibrillation, coronary artery disease, stroke subtype, revascularization therapy, total cholesterol, haemoglobin, and infarct volume.

[c]Patients whose admission NIHSS was 0 (n = 52) were excluded from the analysis.

[d]Patients whose discharge mRS score was 0 (n = 56) or 6 (n = 5) were excluded from the analysis.

[e]Patients whose 3-month mRS score was 0 (n = 69) or 6 (n = 21) were excluded from the analysis. Of these patients, those who were lost to follow-up at 1 year after the index stroke (n = 19) were also excluded from the analysis.

[f]Patients lost to follow-up at 1 year after the index stroke (n = 20) were excluded from the analysis.

CI, confidence interval; END, early neurological deterioration; mRS, modified Rankin Scale; NIHSS, National Institutes of Health Stroke Scale; OR, odds ratio; TMT, temporal muscle thickness.

Supplementary Table 4. Sensitivity Analyses: Clinical Outcomes by TMT / Height^2 in Patients with Acute Ischaemic Stroke[a]

	Low TMT/height^2 Group (n = 150)	High TMT/height^2 Group (n = 450)	P
Dysphagia	57 (38.0)	88 (19.6)	<0.001
END	41 (27.3)	65 (14.4)	<0.001
Stroke recurrence	1 (0.7)	2 (0.4)	>0.99
Stroke progression	31 (20.7)	52 (11.6)	0.005
Symptomatic haemorrhagic Transformation	2 (1.3)	3 (0.7)	0.60
Others	3 (2.0)	1 (0.2)	0.050
Unknown	4 (2.7)	6 (1.3)	0.28
TIA	0 (0.0)	1 (0.2)	>0.99
In-hospital recovery[b]	49/139 (35.3)	188/409 (46.0)	0.03
Post-discharge recovery[c]	37/139 (26.6)	132/400 (33.0)	0.16
Chronic recovery[d]	31/123 (25.2)	100/368 (27.2)	0.67
3-mo mRS ≥3	92 (61.3)	149 (33.1)	<0.001
3-mo mRS ≥4	55 (36.7)	62 (13.8)	<0.001
1-y mRS ≥3[e]	79/139 (56.8)	129/441 (29.3)	<0.001
1-y mRS ≥4[e]	48/139 (34.5)	69/441 (15.7)	<0.001

Data are presented as number (percentage).

[a]Patients were divided into low and high TMT/height^2 groups by using the 25th percentile of TMT/height^2 as the cut-off point.

[b]Patients whose admission NIHSS was 0 (n = 52) were excluded from the analysis.

[c]Patients whose discharge mRS score was 0 (n = 56) or 6 (n = 5) were excluded from the analysis.

[d]Patients whose 3-month mRS score was 0 (n = 69) or 6 (n = 21) were excluded from the analysis. Of these patients, those who were lost to follow-up at 1 year after the index stroke (n = 19) were also excluded from the analysis.

[e]Patients lost to follow-up at 1 year after the index stroke (n = 20) were excluded from the analysis.

END, early neurological deterioration; mRS, modified Rankin Scale; NIHSS, National Institutes of Health Stroke Scale; TIA, transient ischaemic attack; TMT, temporal muscle thickness.

Supplementary Table 5. Sensitivity Analyses: Logistic Regression Analyses to Assess the Relationships between Low TMT / Height^2 and Outcomes in Patients with Acute Ischaemic Stroke[a]

	Univariable analysis : OR (95% CI)	*P*	Multivariable analysis : OR (95% CI)[b]	*P*
Dysphagia	2.52 (1.68-3.78)	<0.001	2.12 (1.23-3.65)	0.007
END	2.23 (1.43-3.48)	<0.001	2.36 (1.41-3.93)	0.001
In-hospital recovery[c]	0.64 (0.43-0.95)	0.03	0.70 (0.46-1.09)	0.12
Post-discharge recovery[d]	0.74 (0.48-1.13)	0.16	0.85 (0.53-1.36)	0.49
Chronic recovery[e]	0.90 (0.57-1.44)	0.67	1.17 (0.70-1.97)	0.54
3-mo mRS ≥3	3.20 (2.19-4.70)	<0.001	3.36 (2.01-5.62)	<0.001
3-mo mRS ≥4	3.62 (2.36-5.55)	<0.001	3.77 (2.06-6.90)	<0.001
1-y mRS ≥3[f]	3.18 (2.15-4.72)	<0.001	2.98 (1.78-4.98)	<0.001
1-y mRS ≥4[f]	2.84 (1.84-4.39)	<0.001	1.99 (1.10-3.59)	0.02

[a]Low TMT/height^2 was defined as below the 25th percentile of TMT/height^2.

[b]Data were adjusted for age, sex, admission NIHSS, body mass index, pre-stroke mRS, previous history of stroke, hypertension, diabetes, hyperlipidaemia, smoking, atrial fibrillation, coronary artery disease, stroke subtype, revascularization therapy, total cholesterol, haemoglobin, and infarct volume.

[c]Patients whose admission NIHSS was 0 (*n* = 52) were excluded from the analysis.

[d]Patients whose discharge mRS score was 0 (*n* = 56) or 6 (*n* = 5) were excluded from the analysis.

[e]Patients whose 3-month mRS score was 0 (*n* = 69) or 6 (*n* = 21) were excluded from the analysis. Of these patients, those who were lost to follow-up at 1 year after the index stroke (*n*

= 19) were also excluded from the analysis.

[f]Patients lost to follow-up at 1 year after the index stroke (*n* = 20) were excluded from the analysis.

CI, confidence interval; END, early neurological deterioration; mRS, modified Rankin Scale; NIHSS, National Institutes of Health Stroke Scale; OR, odds ratio; TMT, temporal muscle thickness.

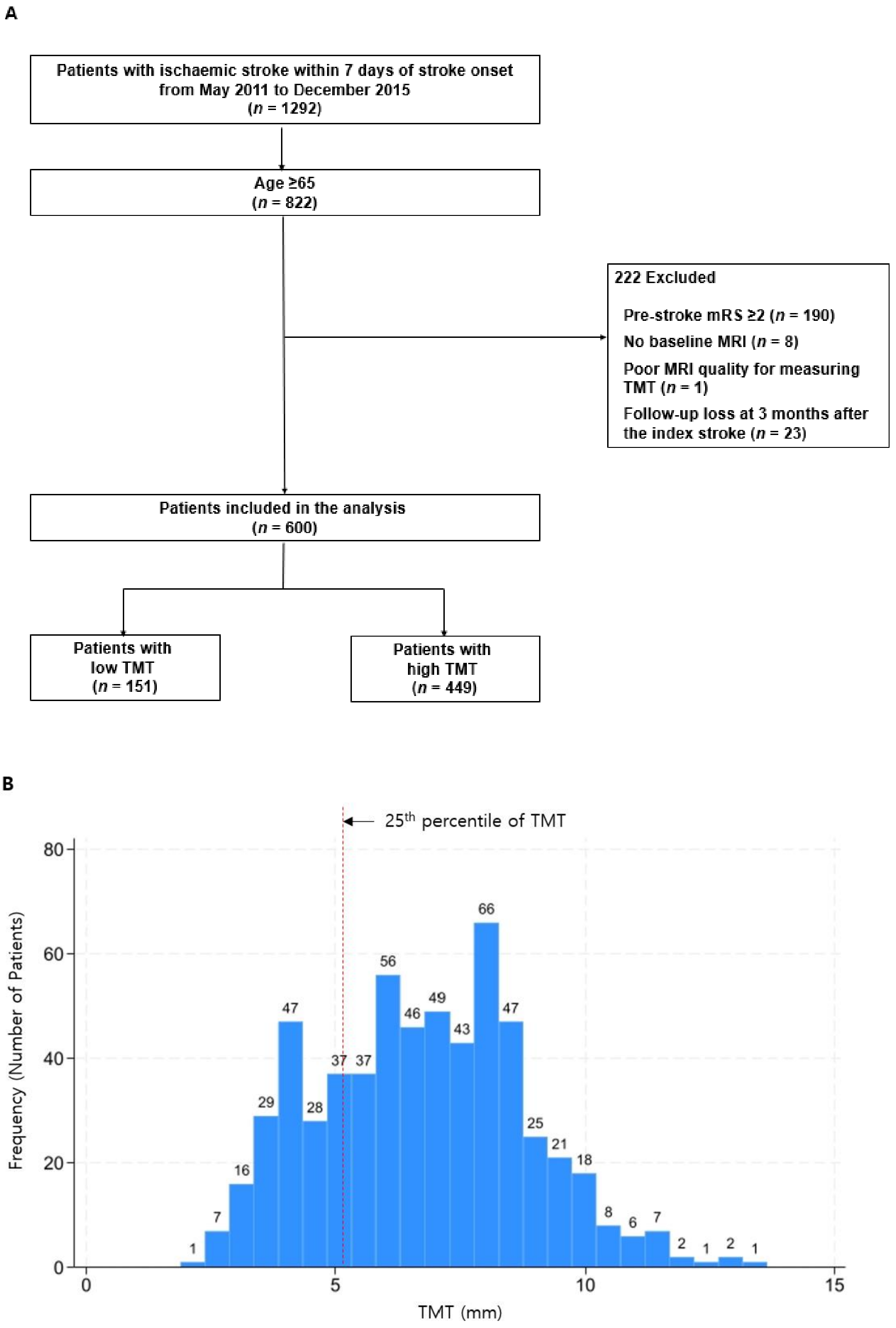

Supplementary Figure 1. (A) Study flowchart and (B) histogram of TMT.

Patients were dichotomised into low and high TMT groups at the 25th percentile cut-off point (5.1 mm).

mRS, modified Rankin Scale; TMT, temporal muscle thickness.

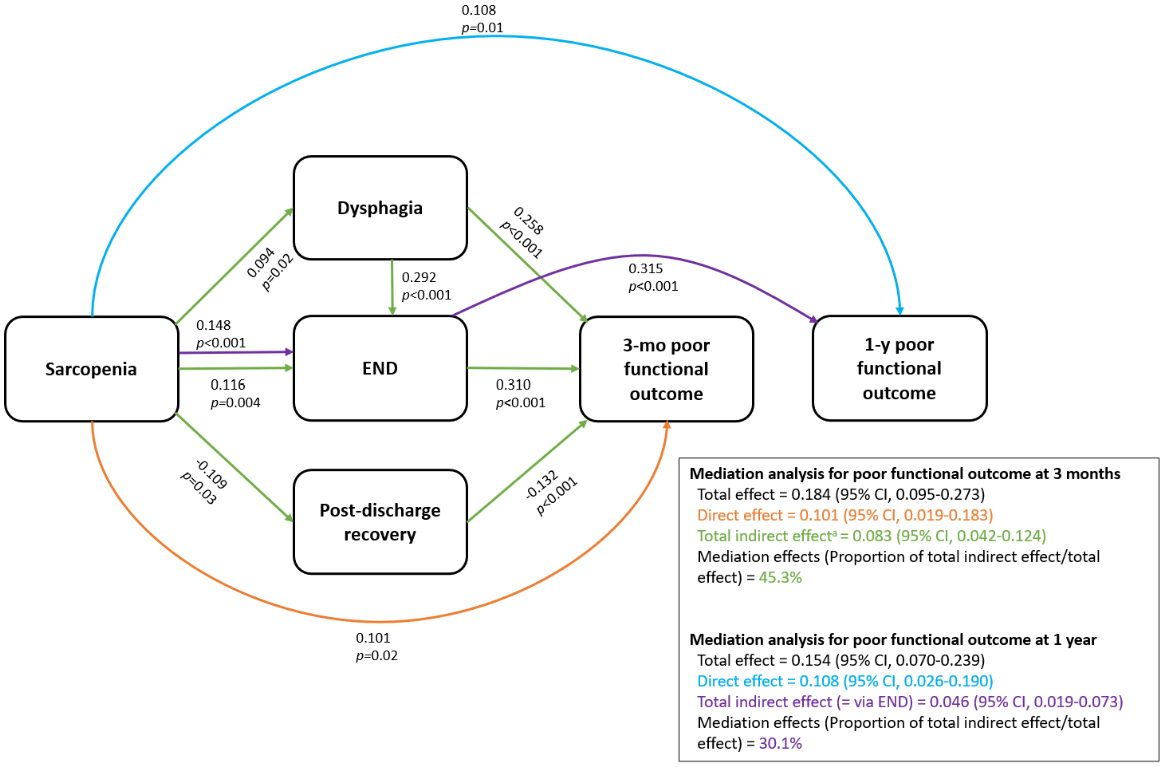

Supplementary Figure 2. Mediation analysis for pathways from sarcopenia to poor functional outcomes (mRS ≥3) at 3 months and 1 year.

Dysphagia, END, and post-discharge recovery mediate 3-month poor functional outcome (green arrows; $n = 539$). END also mediates 1-year outcome (purple arrows; $n = 580$). Orange and blue arrows indicate direct effects on 3-month and 1-year outcomes, respectively. A beta coefficient and a P-value are provided for each pathway.

[a]Note that the total indirect effect sarcopenia had on the 3-month outcome comprised the combined mediation effects of dysphagia, either directly or through END; END; and post-discharge recovery.

END, early neurological deterioration; mRS, modified Rankin Scale.

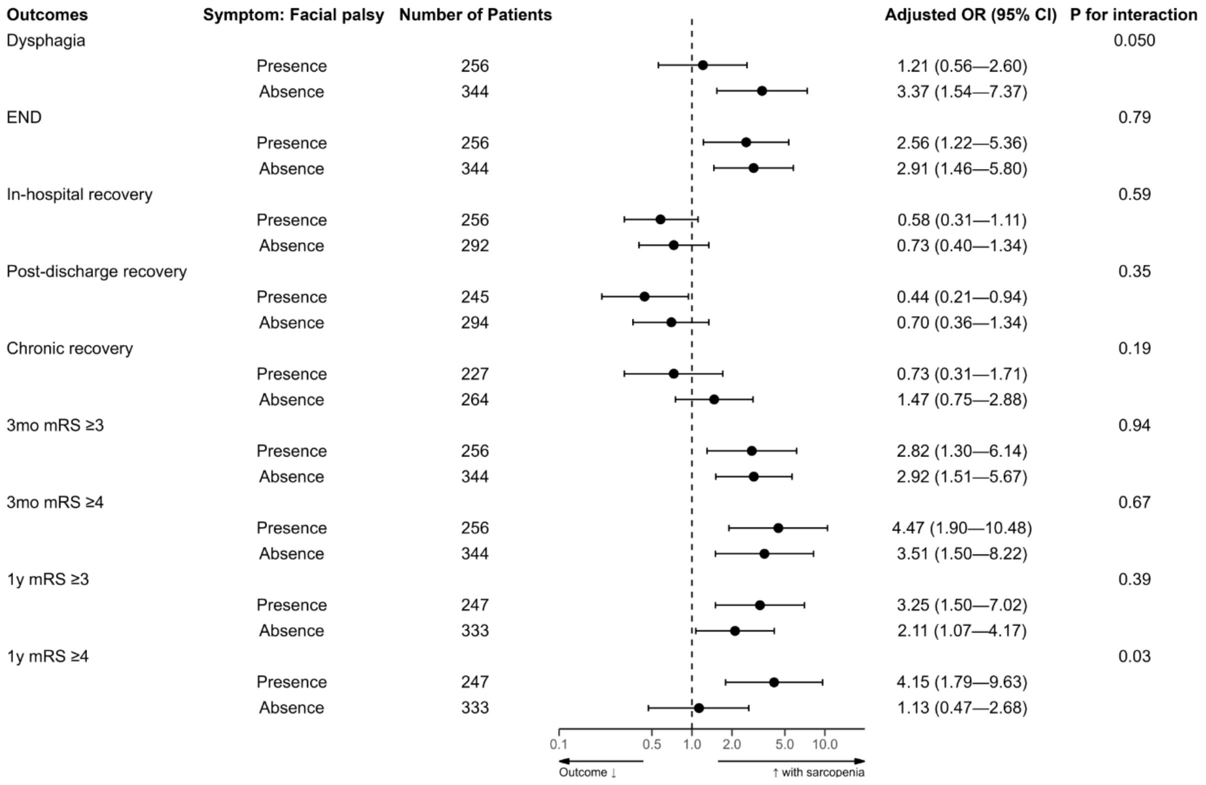

Supplementary Figure 3. Associations between low TMT and stroke outcomes in elderly AIS patients with vs. without facial palsy.

Multivariable logistic regression analyses were performed, including an interaction term between low temporal muscle thickness (TMT) and the presence (vs. absence) of facial palsy, defined as a facial palsy score ≥1 on NIHSS score item 4.

AIS, acute ischaemic stroke; CI, confidence interval; END, early neurological deterioration; mRS, modified Rankin Scale; NIHSS, National Institutes of Health Stroke Scale; OR, odds ratio.

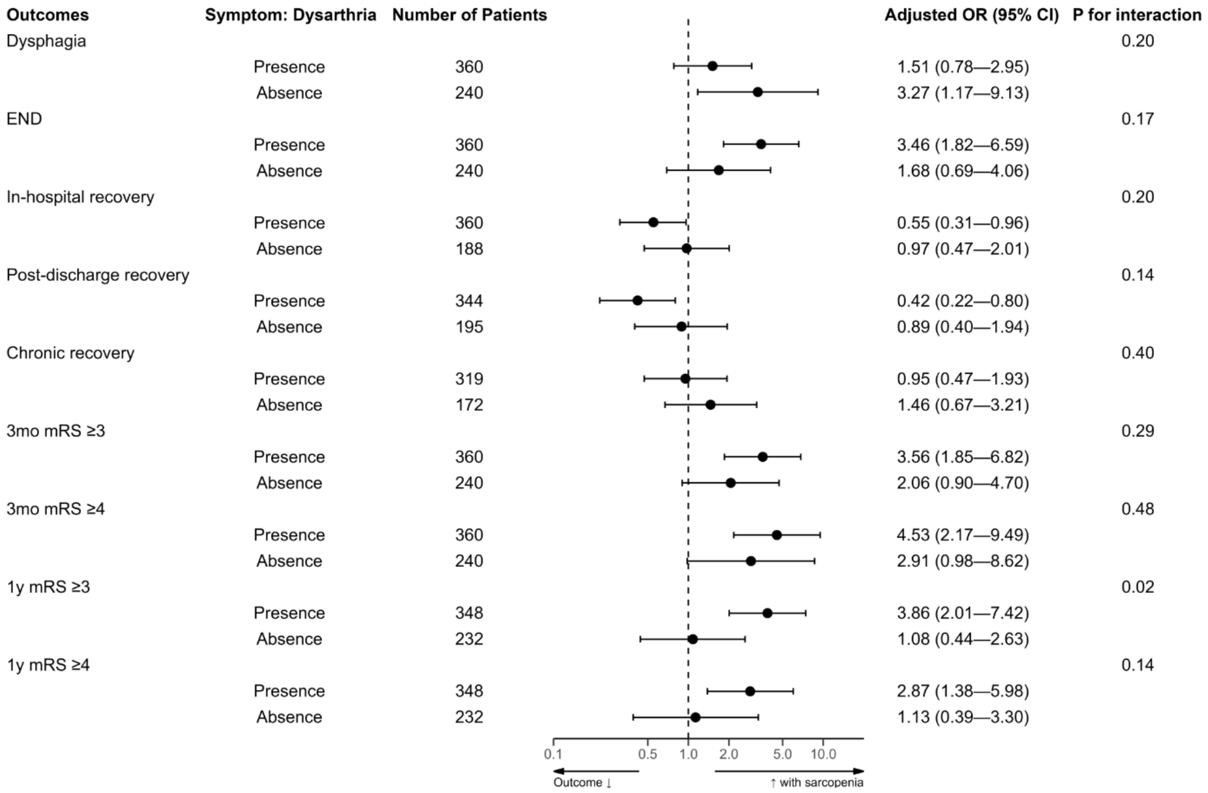

Supplementary Figure 4. Associations between low TMT and stroke outcomes in elderly AIS patients with vs. without dysarthria.

Multivariable logistic regression analyses were performed, including an interaction term between low temporal muscle thickness (TMT) and the presence (vs. absence) of dysarthria, defined as a dysarthria score ≥1 on NIHSS score item 10.

AIS, acute ischaemic stroke; CI, confidence interval; END, early neurological deterioration; mRS, modified Rankin Scale; NIHSS, National Institutes of Health Stroke Scale; OR, odds ratio.

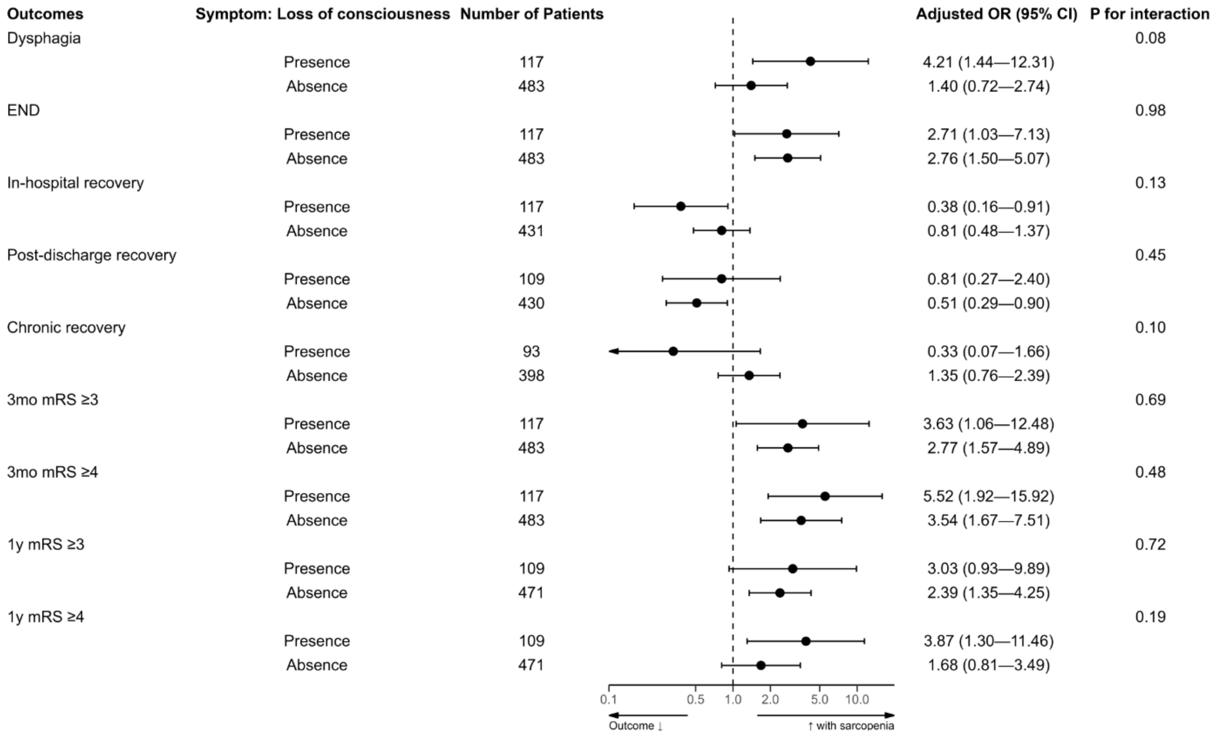

Supplementary Figure 5. Associations between low TMT and stroke outcomes in elderly AIS patients with vs. without loss of consciousness.

Multivariable logistic regression analyses were performed, including an interaction term between low temporal muscle thickness (TMT) and the presence (vs. absence) of loss of consciousness, defined as a loss of consciousness score ≥1 on NIHSS score items 1a-c.

AIS, acute ischaemic stroke; CI, confidence interval; END, early neurological deterioration; mRS, modified Rankin Scale; NIHSS, National Institutes of Health Stroke Scale; OR, odds ratio.

Outcomes	Symptom: Best gaze	Number of Patients	Adjusted OR (95% CI)	P for interaction
Dysphagia				0.36
	Presence	82	1.09 (0.29—4.14)	
	Absence	518	2.10 (1.14—3.88)	
END				0.48
	Presence	82	4.05 (1.27—13.00)	
	Absence	518	2.60 (1.45—4.64)	
In-hospital recovery				0.89
	Presence	82	0.74 (0.26—2.07)	
	Absence	466	0.68 (0.41—1.13)	
Post-discharge recovery				0.37
	Presence	79	0.93 (0.29—3.01)	
	Absence	460	0.53 (0.30—0.92)	
Chronic recovery				0.10
	Presence	72	0.21 (0.02—1.80)	
	Absence	419	1.33 (0.76—2.34)	
3mo mRS ≥3				0.97
	Presence	82	2.92 (0.56—15.34)	
	Absence	518	2.82 (1.62—4.91)	
3mo mRS ≥4				0.15
	Presence	82	1.59 (0.42—6.04)	
	Absence	518	4.61 (2.29—9.27)	
1y mRS ≥3				0.59
	Presence	79	3.70 (0.77—17.73)	
	Absence	501	2.36 (1.35—4.12)	
1y mRS ≥4				0.37
	Presence	79	3.69 (0.95—14.38)	
	Absence	501	1.88 (0.95—3.70)	

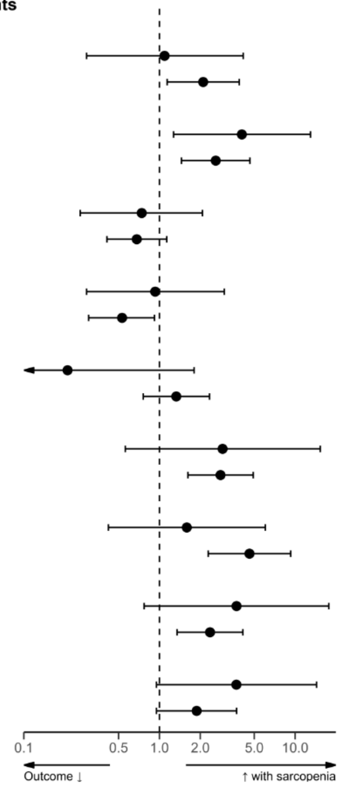

Supplementary Figure 6. Associations between low TMT and stroke outcomes in elderly AIS patients with vs. without gaze abnormalities.

Multivariable logistic regression analyses were performed, including an interaction term between low temporal muscle thickness (TMT) and the presence (vs. absence) of gaze abnormalities, defined as a best gaze score ≥1 on NIHSS score item 2.

AIS, acute ischaemic stroke; CI, confidence interval; END, early neurological deterioration; mRS, modified Rankin Scale; NIHSS, National Institutes of Health Stroke Scale; OR, odds ratio.

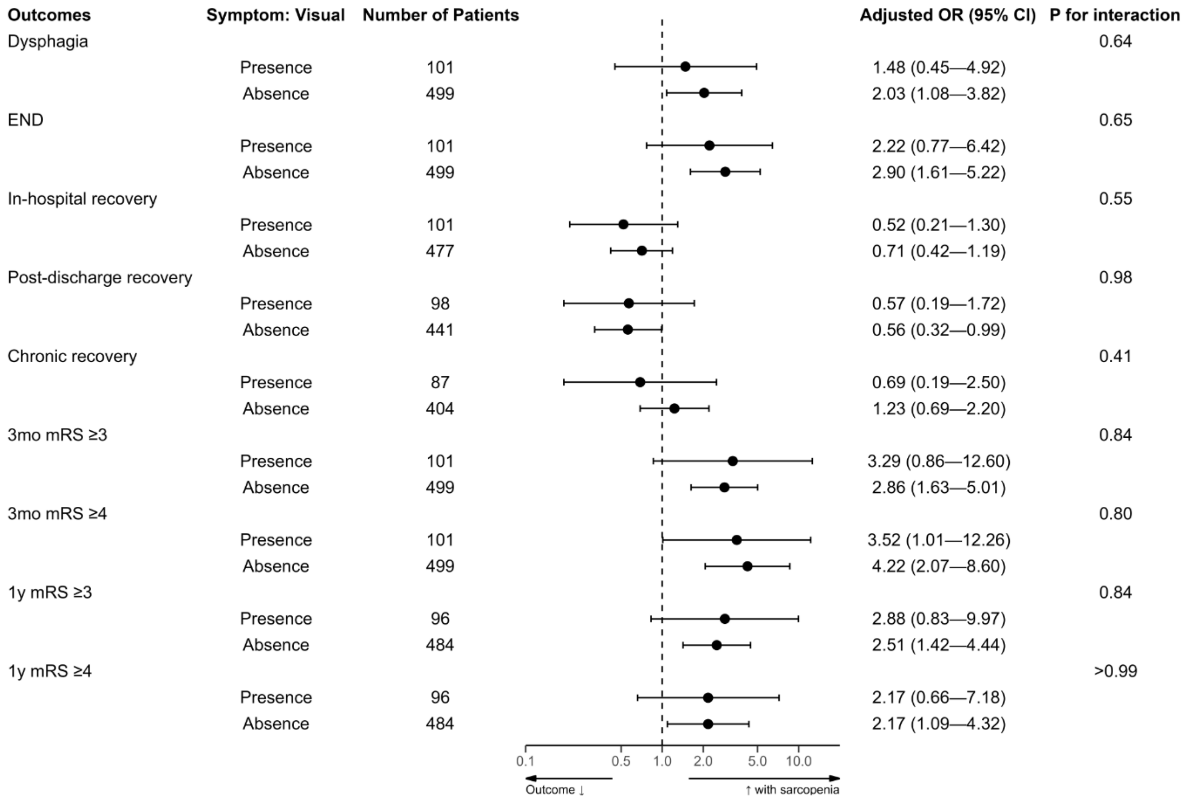

Supplementary Figure 7. Associations between low TMT and stroke outcomes in elderly AIS patients with vs. without abnormalities in either visual acuity or visual field.

Multivariable logistic regression analyses were performed, including an interaction term between low temporal muscle thickness (TMT) and the presence (vs. absence) of visual acuity or visual field, defined as a visual score ≥1 on NIHSS score item 3.

AIS, acute ischaemic stroke; CI, confidence interval; END, early neurological deterioration; mRS, modified Rankin Scale; NIHSS, National Institutes of Health Stroke Scale; OR, odds ratio.

Outcomes	Symptom: Ataxia	Number of Patients	Adjusted OR (95% CI)	P for interaction
Dysphagia				0.26
	Presence	50	6.21 (0.76 —50.63)	
	Absence	550	1.78 (0.98 —3.23)	
END				0.50
	Presence	50	5.16 (0.80 —33.37)	
	Absence	550	2.67 (1.53 —4.65)	
In-hospital recovery				0.07
	Presence	50	2.30 (0.54 —9.76)	
	Absence	498	0.58 (0.36 —0.94)	
Post-discharge recovery				0.71
	Presence	47	0.72 (0.17 —3.07)	
	Absence	492	0.53 (0.31 —0.92)	
Chronic recovery				0.07
	Presence	39	0.09 (0.004—1.69)	
	Absence	452	1.37 (0.79 —2.35)	
3mo mRS ≥3				0.29
	Presence	50	1.12 (0.18 —7.09)	
	Absence	550	3.16 (1.82 —5.49)	
3mo mRS ≥4				0.81
	Presence	50	3.18 (0.47 —21.75)	
	Absence	550	4.08 (2.10 —7.92)	
1y mRS ≥3				0.77
	Presence	49	1.94 (0.30 —12.69)	
	Absence	531	2.57 (1.49 —4.45)	
1y mRS ≥4				0.26
	Presence	49	7.34 (0.80 —67.33)	
	Absence	531	2.00 (1.05 —3.78)	

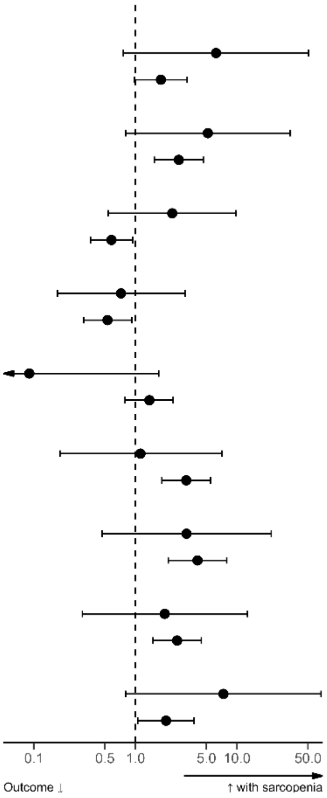

Supplementary Figure 8. Associations between low TMT and stroke outcomes in elderly AIS patients with vs. without ataxia.

Multivariable logistic regression analyses were performed, including an interaction term between low temporal muscle thickness (TMT) and the presence (vs. absence) of ataxia, defined as an ataxia score ≥1 on NIHSS score item 7. A penalised logistic regression model was used for chronic recovery due to rare event rates.

AIS, acute ischaemic stroke; CI, confidence interval; END, early neurological deterioration; mRS, modified Rankin Scale; NIHSS, National Institutes of Health Stroke Scale; OR, odds ratio.

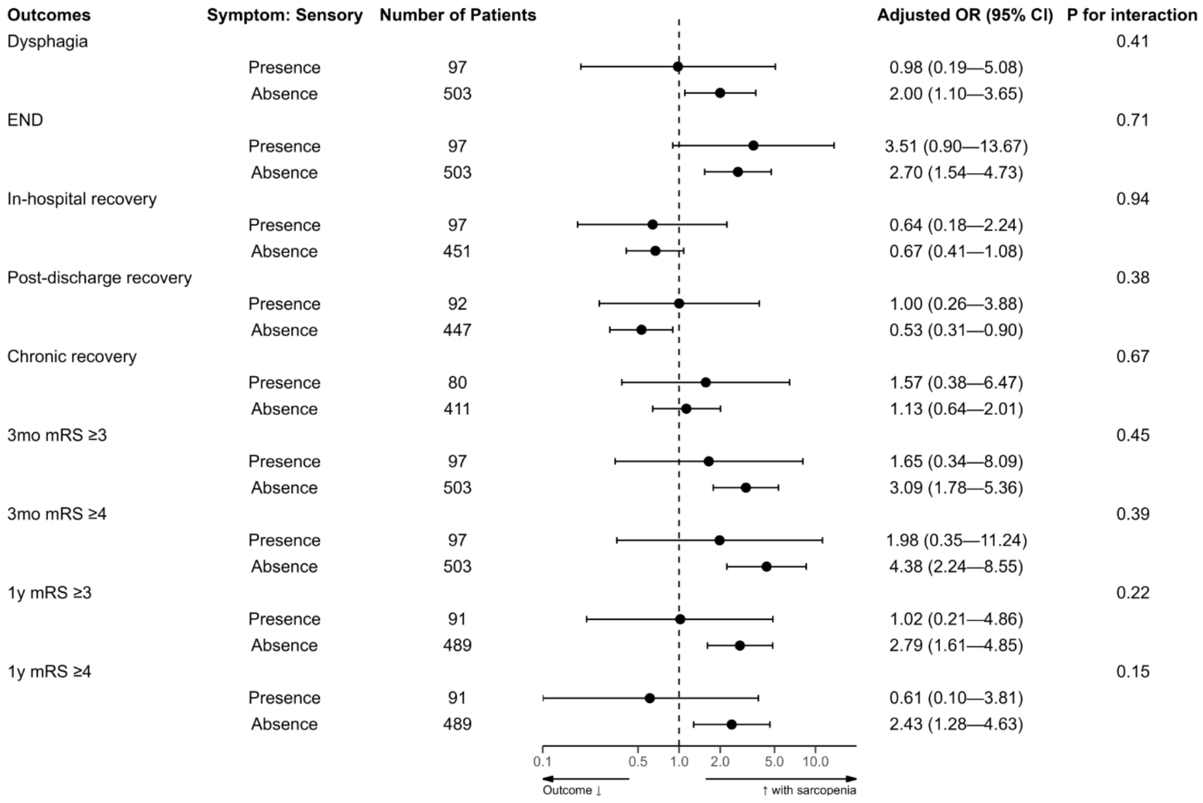

Supplementary Figure 9. Associations between low TMT and stroke outcomes in elderly AIS patients with vs. without sensory abnormalities.

Multivariable logistic regression analyses were performed, including an interaction term between low temporal muscle thickness (TMT) and the presence (vs. absence) of sensory abnormalities, defined as a sensory score ≥1 on NIHSS score item 8.

AIS, acute ischaemic stroke; CI, confidence interval; END, early neurological deterioration; mRS, modified Rankin Scale; NIHSS, National Institutes of Health Stroke Scale; OR, odds ratio.

Outcomes	Symptom: Aphasia	Number of Patients	Adjusted OR (95% CI)	P for interaction
Dysphagia				0.34
	Presence	88	3.56 (0.78—16.14)	
	Absence	512	1.62 (0.87—3.01)	
END				0.46
	Presence	88	4.16 (1.22—14.12)	
	Absence	512	2.54 (1.43—4.52)	
In-hospital recovery				0.63
	Presence	88	0.51 (0.17—1.56)	
	Absence	460	0.69 (0.42—1.12)	
Post-discharge recovery				0.29
	Presence	82	1.07 (0.28—4.02)	
	Absence	457	0.50 (0.29—0.87)	
Chronic recovery				0.36
	Presence	71	0.56 (0.10—2.99)	
	Absence	420	1.26 (0.72—2.23)	
3mo mRS ≥3				0.75
	Presence	88	2.16 (0.40—11.73)	
	Absence	512	2.89 (1.66—5.03)	
3mo mRS ≥4				0.93
	Presence	88	3.65 (0.82—16.20)	
	Absence	512	3.92 (1.97—7.80)	
1y mRS ≥3				0.19
	Presence	86	7.33 (1.29—41.52)	
	Absence	494	2.18 (1.24—3.81)	
1y mRS ≥4				0.21
	Presence	86	5.02 (1.09—23.17)	
	Absence	494	1.75 (0.89—3.45)	

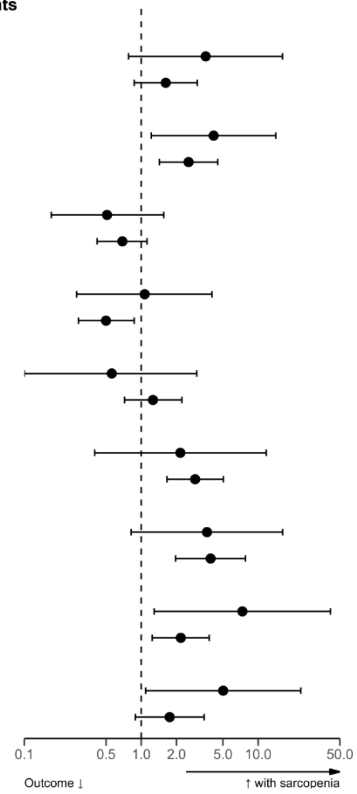

Supplementary Figure 10. Associations between low TMT and stroke outcomes in elderly AIS patients with vs. without aphasia.

Multivariable logistic regression analyses were performed, including an interaction term between low temporal muscle thickness (TMT) and the presence (vs. absence) of aphasia, defined as a best language score ≥1 on NIHSS score item 9.

AIS, acute ischaemic stroke; CI, confidence interval; END, early neurological deterioration; mRS, modified Rankin Scale; NIHSS, National Institutes of Health Stroke Scale; OR, odds ratio.

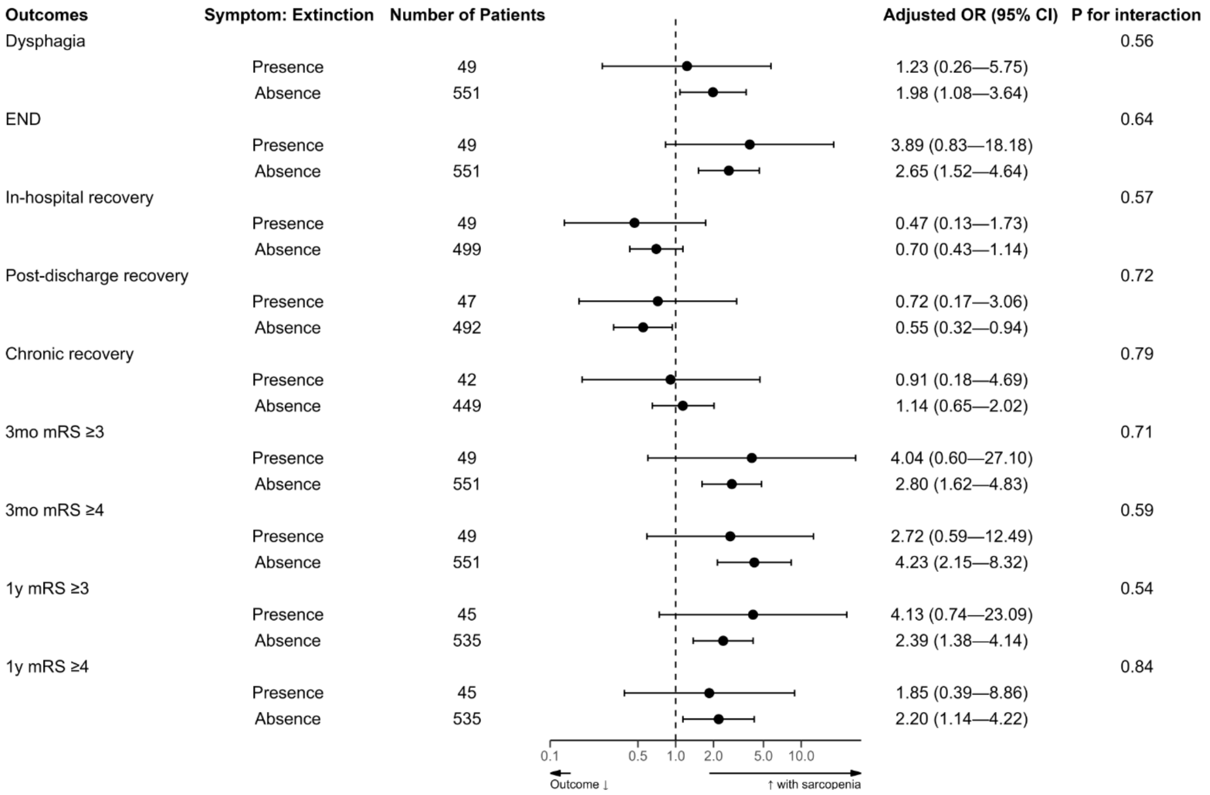

Supplementary Figure 11. Associations between low TMT and stroke outcomes in elderly AIS patients with vs. without extinction and inattention.

Multivariable logistic regression analyses were performed, including an interaction term between low temporal muscle thickness (TMT) and the presence (vs. absence) of extinction and inattention, defined as extinction and inattention score ≥1 on NIHSS score item 11.

AIS, acute ischaemic stroke; CI, confidence interval; END, early neurological deterioration; mRS, modified Rankin Scale; NIHSS, National Institutes of Health Stroke Scale; OR, odds ratio.

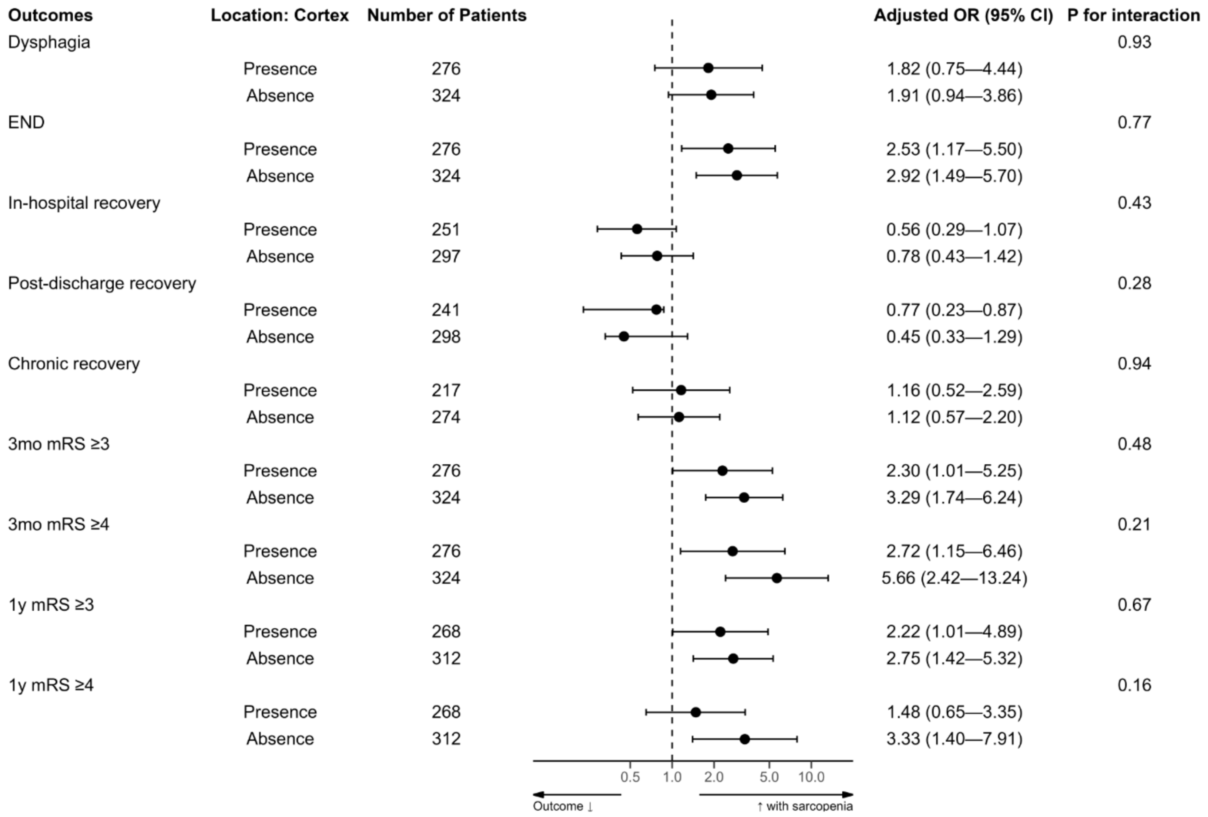

Supplementary Figure 12. Associations between low TMT and stroke outcomes in elderly AIS patients with vs. without cortical lesions.

Multivariable logistic regression analyses were performed, with an interaction term between low temporal muscle thickness (TMT) and the presence (vs. absence) of cortical lesions.

AIS, acute ischaemic stroke; CI, confidence interval; END, early neurological deterioration; mRS, modified Rankin Scale; OR, odds ratio.

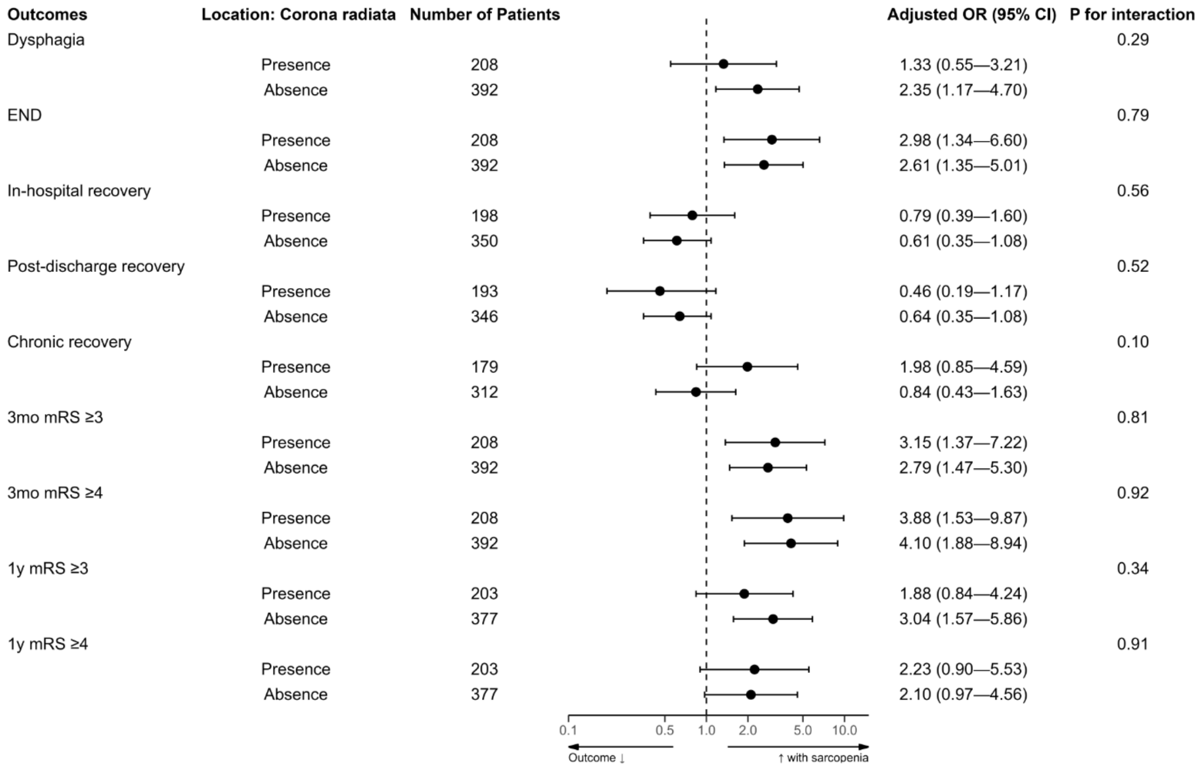

Supplementary Figure 13. Associations between low TMT and stroke outcomes in elderly AIS patients with vs. without corona radiata lesions.

Multivariable logistic regression analyses were performed, with an interaction term between low temporal muscle thickness (TMT) and the presence (vs. absence) of corona radiata lesions.

AIS, acute ischaemic stroke; CI, confidence interval; END, early neurological deterioration; mRS, modified Rankin Scale; OR, odds ratio.

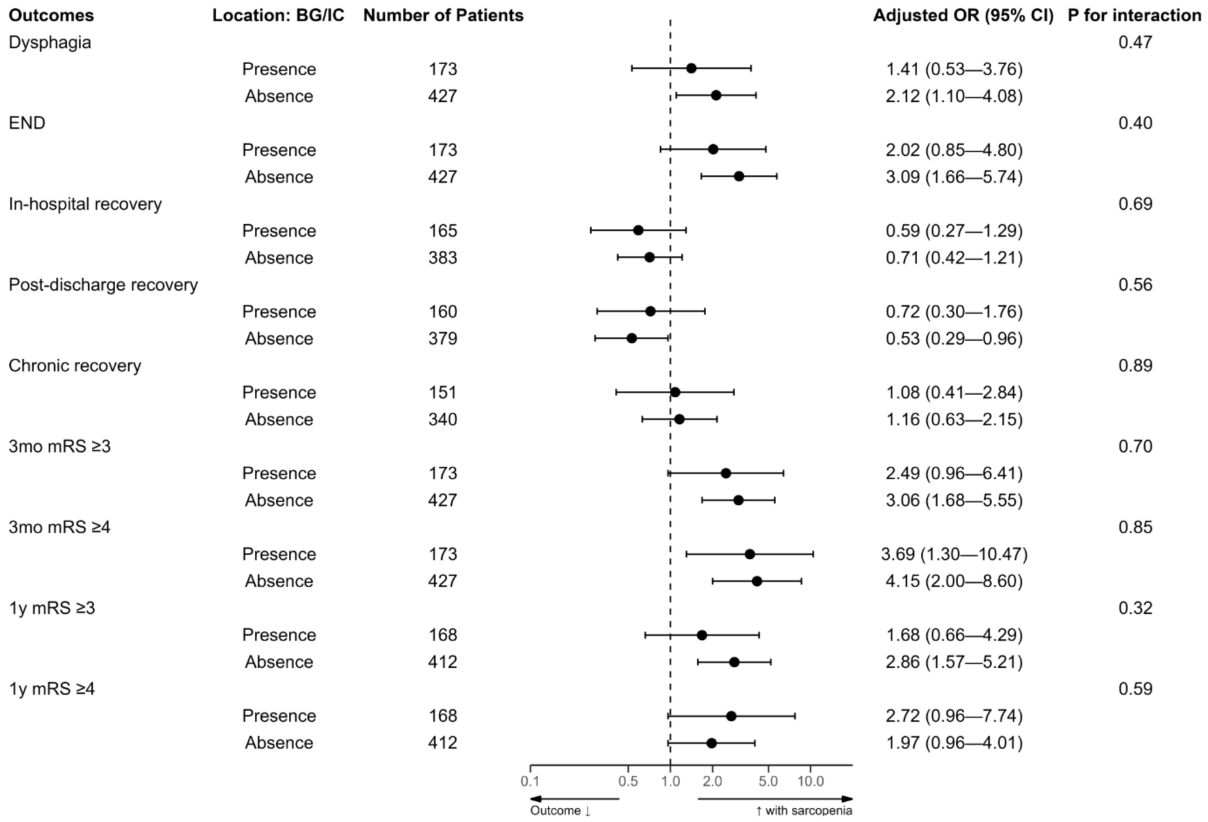

Supplementary Figure 14. Associations between low TMT and stroke outcomes in elderly AIS patients with vs. without basal ganglia or internal capsule lesions.

Multivariable logistic regression analyses were performed, with an interaction term between low temporal muscle thickness (TMT) and the presence (vs. absence) of BG/IC lesions.

AIS, acute ischaemic stroke; BG/IC, basal ganglia/internal capsule; CI, confidence interval; END, early neurological deterioration; mRS, modified Rankin Scale; OR, odds ratio.

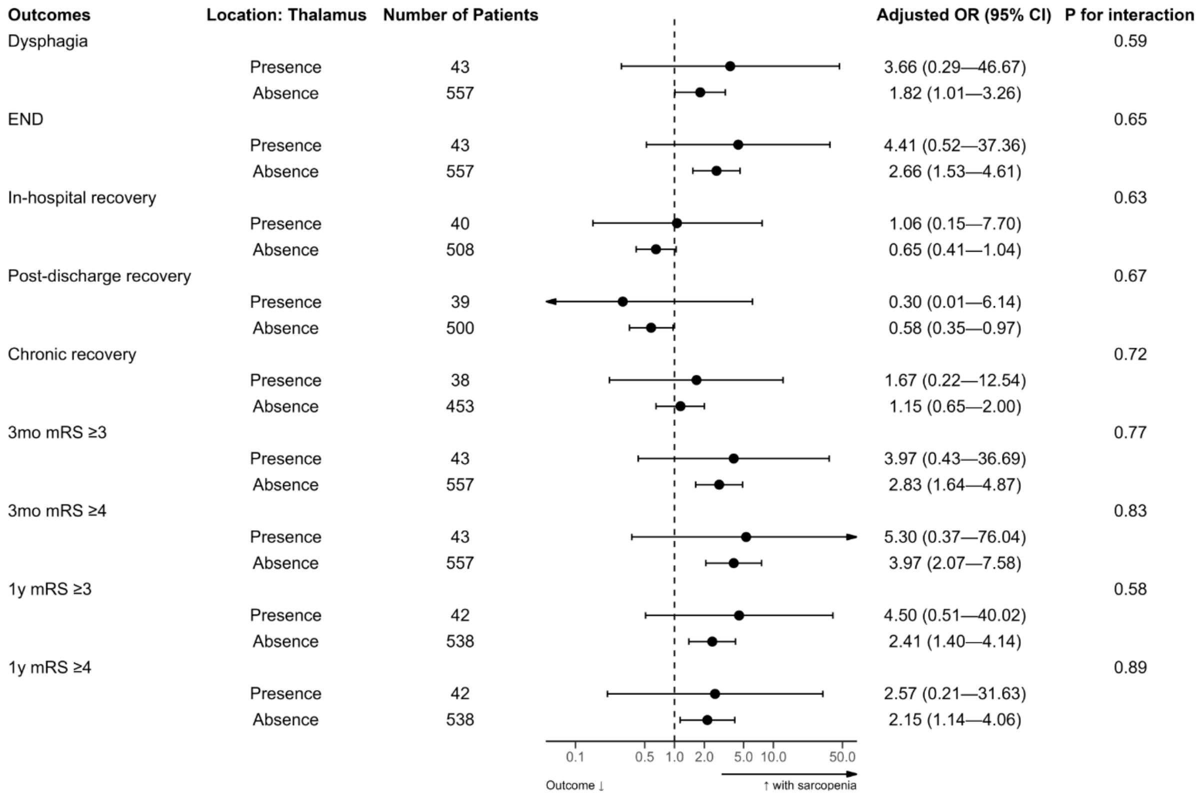

Supplementary Figure 15. Associations between low TMT and stroke outcomes in elderly AIS patients with vs. without thalamic lesions.

Multivariable logistic regression analyses were performed, with an interaction term between low temporal muscle thickness (TMT) and the presence (vs. absence) of thalamic lesions. A penalised logistic regression model was used for post-discharge recovery due to rare event rates.

AIS, acute ischaemic stroke; CI, confidence interval; END, early neurological deterioration; mRS, modified Rankin Scale; OR, odds ratio.

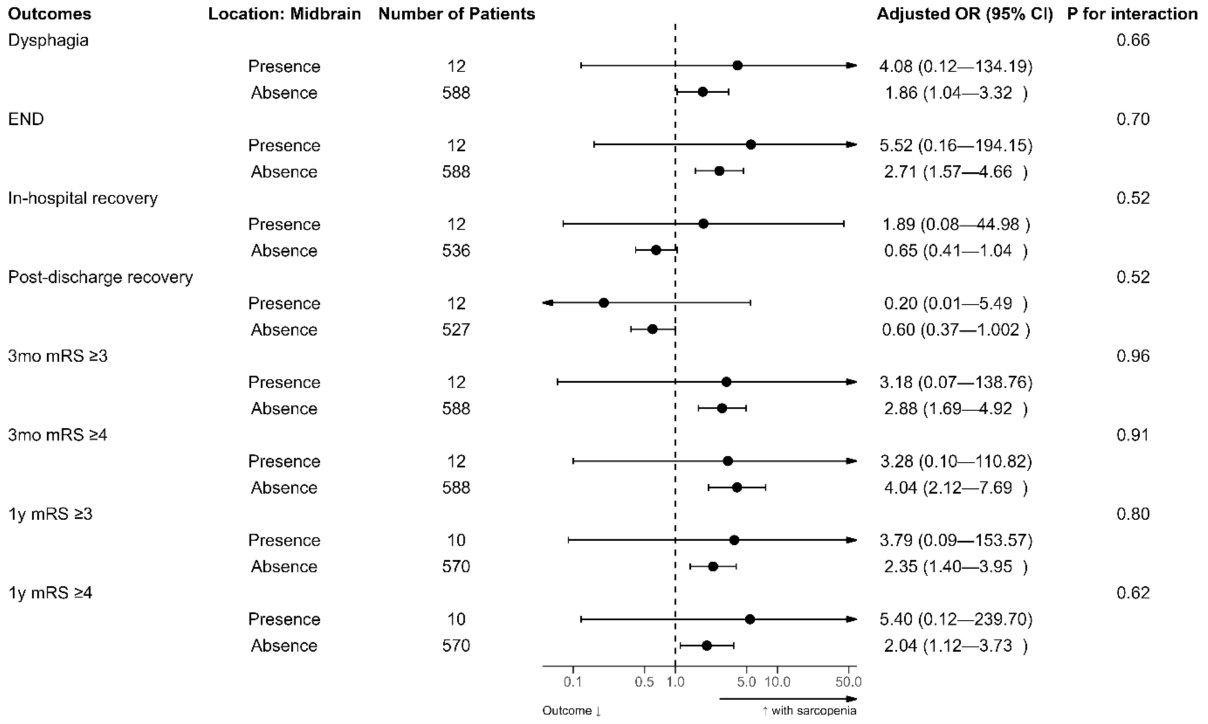

Supplementary Figure 16. Associations between low TMT and stroke outcomes in elderly AIS patients with vs. without midbrain lesions.

Multivariable logistic regression analyses were performed, with an interaction term between low temporal muscle thickness (TMT) and the presence (vs. absence) of midbrain lesions. Penalised logistic regression models were used for post-discharge recovery, 1-year mRS ≥3, and 1-year mRS ≥4 due to rare event rates.

Note that interaction analysis of how the association between low TMT and midbrain lesions affected chronic recovery could not be performed due to a low number of events.

AIS, acute ischaemic stroke; CI, confidence interval; END, early neurological deterioration; mRS, modified Rankin Scale; OR, odds ratio.

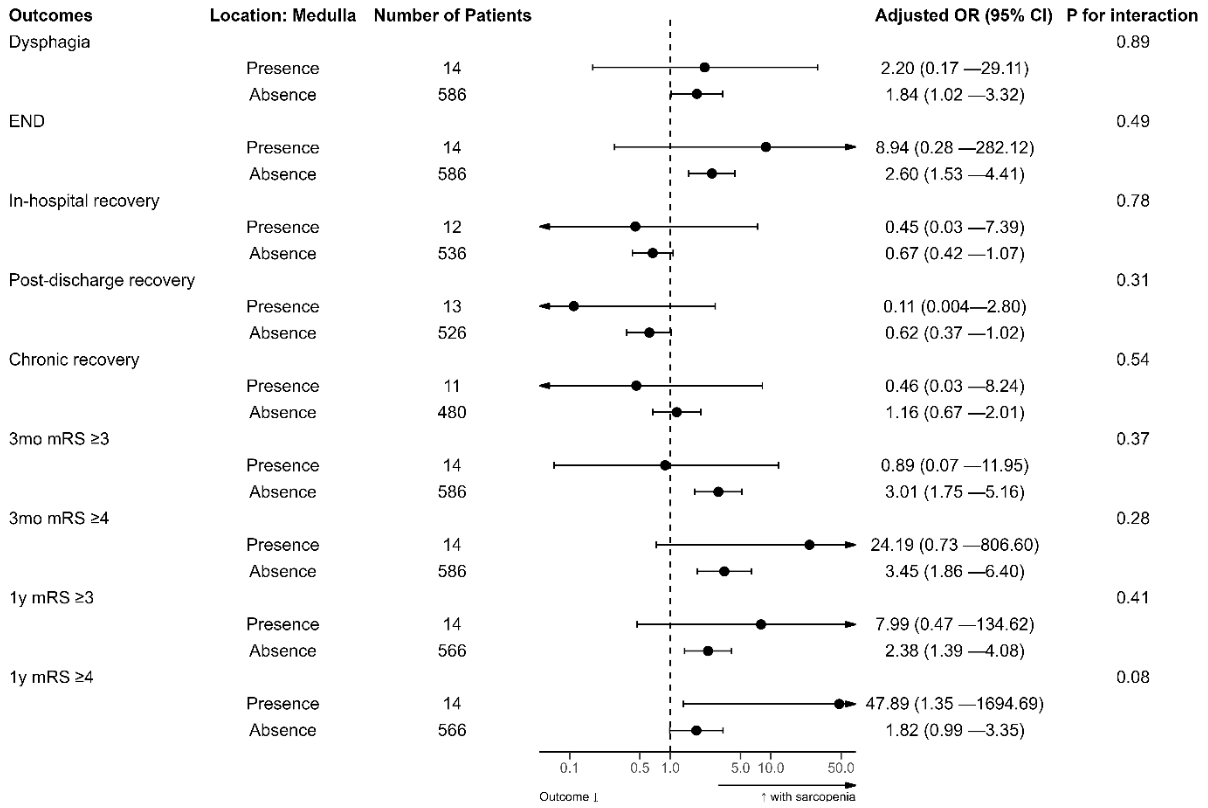

Supplementary Figure 17. Associations between low TMT and stroke outcomes in elderly AIS patients with vs. without medullary lesions.

Multivariable logistic regression analyses were performed, with an interaction term between low temporal muscle thickness (TMT) and the presence (vs. absence) of medullary lesions. Penalised logistic regression models were used for END, post-discharge recovery, 3-months mRS ≥4, and 1-year mRS ≥4 due to rare event rates.

AIS, acute ischaemic stroke; CI, confidence interval; END, early neurological deterioration; mRS, modified Rankin Scale; OR, odds ratio.

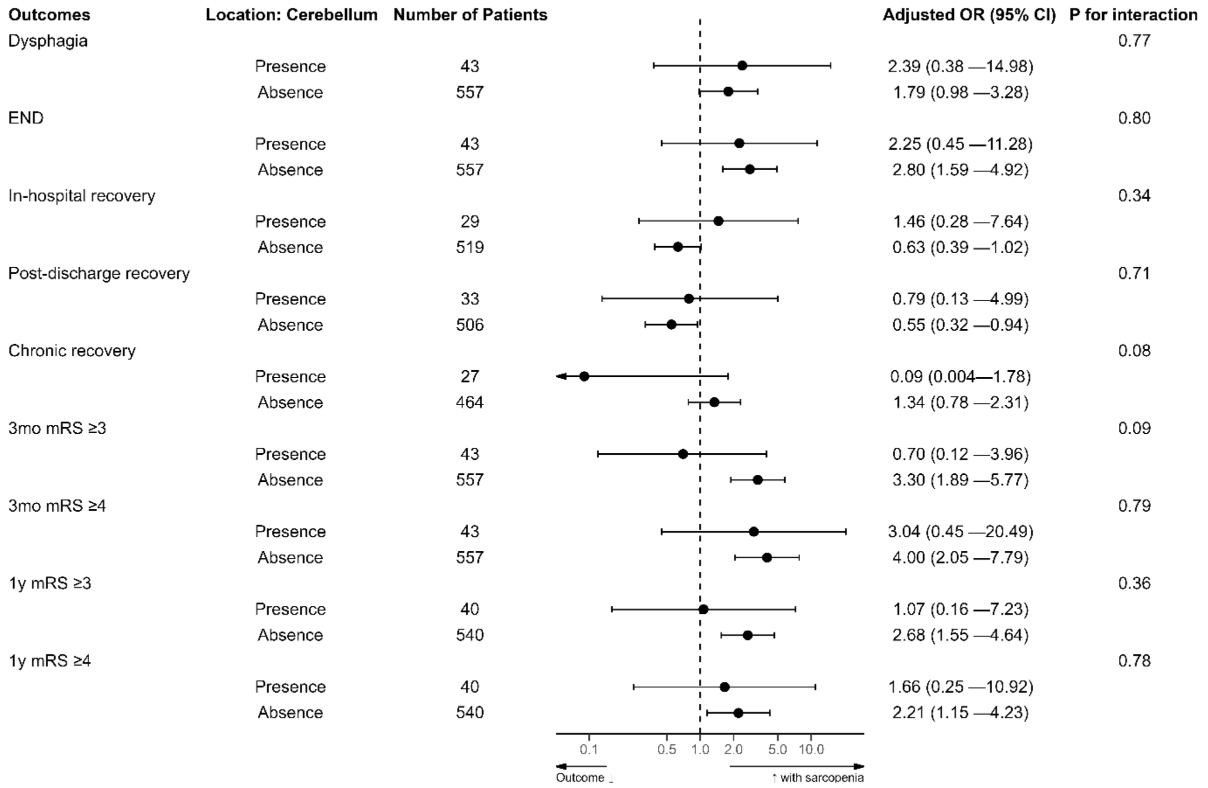

Supplementary Figure 18. Associations between low TMT and stroke outcomes in elderly AIS patients with vs. without cerebellar lesions.

Multivariable logistic regression analyses were performed, with an interaction term between low temporal muscle thickness (TMT) and the presence (vs. absence) of cerebellar lesions. A penalised logistic regression model was used for chronic recovery due to rare event rates.

AIS, acute ischaemic stroke; CI, confidence interval; END, early neurological deterioration; mRS, modified Rankin Scale; OR, odds ratio.

Supplementary References

S1. Ohyama K, Watanabe M, Nosaki Y, Hara T, Iwai K, Mokuno K. Correlation Between Skeletal Muscle Mass Deficit and Poor Functional Outcome in Patients with Acute Ischemic Stroke. *J Stroke Cerebrovasc Dis*. Apr 2020;29(4):104623. doi:10.1016/j.jstrokecerebrovasdis.2019.104623

S2. Abe T, Iwata K, Yoshimura Y*, et al*. Low Muscle Mass is Associated with Walking Function in Patients with Acute Ischemic Stroke. *J Stroke Cerebrovasc Dis*. Nov 2020;29(11):105259. doi:10.1016/j.jstrokecerebrovasdis.2020.105259

S3. Han M, Lim IH, Hong SH, Nam HS, Heo JH, Kim YD. Initial stroke severity and discharge outcome in patients with muscle mass deficit. *Sci Rep*. Jan 22 2024;14(1):1911. doi:10.1038/s41598-024-52381-0

S4. Lee H, Lee IH, Heo J*, et al*. Impact of Sarcopenia on Functional Outcomes Among Patients With Mild Acute Ischemic Stroke and Transient Ischemic Attack: A Retrospective Study. *Front Neurol*. 2022;13:841945. doi:10.3389/fneur.2022.841945

S5. Honma K, Honda Y, Nagase M*, et al*. Impact of skeletal muscle mass on functional prognosis in acute stroke: A cohort study. *J Clin Neurosci*. Jun 2023;112:43-47. doi:10.1016/j.jocn.2023.04.006

S6. Nozoe M, Noguchi M, Kubo H, Kanai M, Shimada S. Association between the coexistence of premorbid sarcopenia, frailty, and disability and functional outcome in older patients with acute stroke. *Geriatr Gerontol Int*. Aug 2022;22(8):642-647. doi:10.1111/ggi.14432

S7. Nozoe M, Kanai M, Kubo H, Yamamoto M, Shimada S, Mase K. Prestroke sarcopenia and functional outcomes in elderly patients who have had an acute stroke: A prospective cohort

study. *Nutrition*. Oct 2019;66:44-47. doi:10.1016/j.nut.2019.04.011

S8. Lee SH, Choi H, Kim KY, Lee HS, Jung JM. Appendicular Skeletal Muscle Mass Associated with Sarcopenia as a Predictor of Poor Functional Outcomes in Ischemic Stroke. *Clin Interv Aging*. 2023;18:1009-1020. doi:10.2147/CIA.S402548

S9. Lin YH, Chung CT, Chen CH*, et al*. Association of temporalis muscle thickness with functional outcomes in patients undergoing endovascular thrombectomy. *Eur J Radiol*. Jun 2023;163:110808. doi:10.1016/j.ejrad.2023.110808

S10. Nozoe M, Kubo H, Kanai M*, et al*. Reliability and validity of measuring temporal muscle thickness as the evaluation of sarcopenia risk and the relationship with functional outcome in older patients with acute stroke. *Clin Neurol Neurosurg*. Feb 2021;201:106444. doi:10.1016/j.clineuro.2020.106444

S11. Tutal Gursoy G, Gorgulu U, Tengirsenk Z, Bektas H. The relationship between temporal muscle thickness and triglyceride glucose index in sarcopenia with mortality and third-month functional outcomes in patients after acute stroke. *Medicine (Baltimore)*. Nov 3 2023;102(44):e35886. doi:10.1097/MD.0000000000035886

S12. Nozoe M, Kanai M, Kubo H, Yamamoto M, Shimada S, Mase K. Prestroke Sarcopenia and Stroke Severity in Elderly Patients with Acute Stroke. *J Stroke Cerebrovasc Dis*. Aug 2019;28(8):2228-2231. doi:10.1016/j.jstrokecerebrovasdis.2019.05.001

S13. Fukuma K, Kamada M, Yamamoto K*, et al*. Pre-existing sarcopenia and swallowing outcomes in acute stroke patients. *Clin Nutr*. Aug 2023;42(8):1454-1461. doi:10.1016/j.clnu.2023.06.012

S14. Sakai K, Katayama M, Nakajima J*, et al*. Temporal muscle thickness is associated with the severity of dysphagia in patients with acute stroke. *Arch Gerontol Geriatr*. Sep-Oct

2021;96:104439. doi:10.1016/j.archger.2021.104439

S15. Song X, Chen X, Bai J, Zhang J. Association between pre-stroke sarcopenia risk and stroke-associated infection in older people with acute ischemic stroke. *Front Med (Lausanne)*. 2023;10:1090829. doi:10.3389/fmed.2023.1090829

S16. Imamura M, Nozoe M, Kubo H, Shimada S. Association between premorbid sarcopenia and neurological deterioration in patients with acute ischemic stroke. *Clin Neurol Neurosurg*. Jan 2023;224:107527. doi:10.1016/j.clineuro.2022.107527

S17. Namgung HG, Hong S, Choi YA. Association of Temporalis Muscle Mass with Early Cognitive Impairment in Older Patients with Acute Ischemic Stroke. *J Clin Med*. Jun 15 2023;12(12)doi:10.3390/jcm12124071

S18. Li YX, Hou J, Liu WY. Long-term prognostic significance of sarcopenia in acute ischemic stroke. *Medicine (Baltimore)*. Aug 26 2022;101(34):e30031. doi:10.1097/MD.0000000000030031
